# The Role of Selected Natural Biomolecules in Sperm Production and Functionality

**DOI:** 10.3390/molecules26175196

**Published:** 2021-08-27

**Authors:** Eva Tvrdá, Filip Benko, Tomáš Slanina, Stefan S. du Plessis

**Affiliations:** 1 Department of Animal Physiology, Faculty of Biotechnology and Food Sciences, Slovak University of Agriculture, Tr. A. Hlinku 2, 94976 Nitra, Slovakia; filip.benko276@gmail.com (F.B.); tomas.slanina@uniag.sk (T.S.); 2 Department of Basic Sciences, College of Medicine, Mohammed Bin Rashid University of Medicine and Health Sciences, Dubai 505055, United Arab Emirates; stefan.duplessis@mbru.ac.ae

**Keywords:** natural biomolecules, polyphenols, flavonoids, curcumin, lycopene, phytoestrogens, spermatozoa, male fertility, spermatogenesis

## Abstract

Emerging evidence from in vivo as well as in vitro studies indicates that natural biomolecules may play important roles in the prevention or management of a wide array of chronic diseases. Furthermore, the use of natural compounds in the treatment of male sub- or infertility has been proposed as a potential alternative to conventional therapeutic options. As such, we aimed to evaluate the effects of selected natural biomolecules on the sperm production, structural integrity, and functional activity. At the same time, we reviewed their possible beneficial or adverse effects on male reproductive health. Using relevant keywords, a literature search was performed to collect currently available information regarding molecular mechanisms by which selected natural biomolecules exhibit their biological effects in the context of male reproductive dysfunction. Evidence gathered from clinical trials, in vitro experiments and in vivo studies suggest that the selected natural compounds affect key targets related to sperm mitochondrial metabolism and motion behavior, oxidative stress, inflammation, DNA integrity and cell death. The majority of reports emphasize on ameliorative, stimulating and protective effects of natural biomolecules on the sperm function. Nevertheless, possible adverse and toxic behavior of natural compounds has been indicated as well, pointing out to a possible dose-dependent impact of natural biomolecules on the sperm survival and functionality. As such, further research leading to a deeper understanding of the beneficial or adverse roles of natural compounds is necessary before these can be employed for the management of male reproductive dysfunction.

## 1. Introduction

According to the World Health Organization (WHO), infertility is defined as a disease of the male or female reproductive system characterized by the inability to accomplish a pregnancy following at least 12 months of regular unprotected sexual intercourse [1]. While it is estimated that between 48 million couples and 186 million individuals live with infertility globally, the prevalence of reproductive dysfunction varies between regions and countries [1,2,3].

According to Agarwal et al. [3], the distribution of infertility due to male factors oscillates between 20% and 70%, while in general, male reproductive failure accounts for half of the couple’s infertility. It has been estimated that male infertility factor is solely present in 30% of couples [4].

The main causes of male sub- or infertility encompass genetic factors (including chromosomal abnormalities, numerical aberrations, protamine deficiencies or deletions in the AZF region) [5], disorders of the endocrine system (such as hypothyroidism or Kallmann’s syndrome) [6], medical conditions related to the male reproductive system (such as varicocele, cryptorchidism, erectile dysfunction) or systemic diseases affecting male reproduction (diabetes, cystic fibrosis or tumor development) [7], lifestyle choices (such as poor diet, tobacco smoking, alcohol consumption or drug abuse) [8], urogenital infections [9] or exposure to environmental pollutants (such as heavy metals, endocrine disruptors or radiation) [10,11]. Idiopathic male infertility is acknowledged after excluding all other known factors contributing to infertility, and accounts for up to 25% of male reproductive dysfunction [12].

Poor semen quality and abnormal sperm structure or function by and large constitute a predominant presentation of male infertility and are among the major reasons why couples seek the services of assisted reproduction [12]. The pathological processes that may lead to unexplained aberrations in the sperm behavior are complex and often poorly understood [4]. Generally speaking, contemporary male infertility management and/or treatment has focused on three predominant molecular mechanisms which have been associated with: (a) lipid peroxidation, with subsequent alterations to the membrane fluidity and permeability, and leading to a decreased sperm motility and the ability of male gametes to interact with the ovum; (b) protein modifications which may cause a reduced sperm metabolism and ATP synthesis; and (c) an increased damage to sperm DNA [13].

Various medicaments or nutritional supplements have been studied as potential remedies to improve poor-quality semen in men diagnosed with sub- or infertility. While antioxidants such as vitamin C or E, coenzyme Q10, L-carnitine, selenium or zinc have yielded positive outcomes in several reports, other studies conclude that the above-mentioned molecules had little to no effect on the sperm quality in infertile subjects [14]. In the meantime, it has been revealed that traditional therapies based on the administration of gonadotropins and androgen agonists, or antagonists are not effective for the treatment of men presenting with a substandard semen quality [15,16]. As such, management of these patients remains challenging to this date, which is why 30–40% of sub- or infertile men seek alternative treatment options such as nutraceuticals and functional foods, groceries fortified with plant extracts, dietary supplements, natural health products as well as traditional ethnopharmacological remedies and phytomedicines [17,18].

Natural biomolecules, such as polyphenols or flavonoids have garnered substantial attention from the scientific community as well as public media. This spotlight may be primarily attributed to an emerging evidence emphasizing on beneficial roles of natural biomolecules in the prevention of a vast array of ailments such as diabetes, cardiovascular and neurodegenerative diseases, malignancies, or inflammatory processes [19,20]. Natural biomolecules are considered to be agents that can target multiple cellular pathways, and unlike traditional medicaments present with potent pharmacological properties and intrinsic biologic activity, extraordinary chemical diversity and structural complexity [21].

In the meantime, it has been suggested that administration of natural biomolecules has been shown to have a positive impact male infertility [18,22,23,24,25,26]. A number of in vivo as well as in vitro studies have reported that active components isolated from natural resources could enhance blood circulation in the male reproductive system and support the synthesis and secretion of androgens [27,28]. Animal and human reports also suggest that natural biomolecules may play important roles in the enhancement of semen quality, including sperm concentration, motility, vitality, and structural integrity [29,30,31]. Nevertheless, despite numerous beneficial effects observed in the available literature, there are still concerns associated with a certain dichotomy of major bioactive biomolecules, and subsequent potential hazards associated with an excessive consumption of natural compounds [18,32,33].

The aim of this review is to evaluate the effects of selected natural biomolecules on the sperm production and function, and to propose the mechanisms by which they could act on male gametes. Furthermore, we aim to review their possible beneficial and/or adverse effects on male reproductive health.

## 2. Resveratrol

Resveratrol (3,5,40-trihydroxistilbene; RES) is a polyphenol found in a wide array of dietary sources such as grapes, peanuts, berries, pistachios, plums, and red wine. The molecule is a phytoalexin, as its inherent function is to offer protection to the producer against exogenous stress factors (UV radiation, ozone, injury, or fungal infection). Because of its structural similarities with estradiol or diethylstilbestrol and its ability to modulate estrogen-sensitive systems, RES is defined as a phytoestrogen [34,35].

Being probably the most studied biomolecule of recent decades, RES has attracted widespread scientific attention because of the “French paradox” hypothesis, which associates low incidence of cardiovascular diseases and a long-life expectancy of French people to a moderate consumption of red wine despite a diet containing meals with high amounts of saturated fat [23,34,35].

Currently available literature suggests that RES presents with a wide array of properties that may be useful in the prevention and improvement of a variety of health issues including obesity, diabetes, atherosclerosis, hypertension, malignant and neurodegenerative diseases. Furthermore, the biomolecule presents with anti-inflammatory, antimicrobial, anti-aging, and antioxidant effects [34,35]. Resveratrol is also the most potent bioactive substance that activates sirtuin 1 (SIRT 1), the most-conserved mammalian NAD^+^-dependent protein, which may account for its numerous metabolic benefits in animals as well as in humans [36].

**Figure 1:**
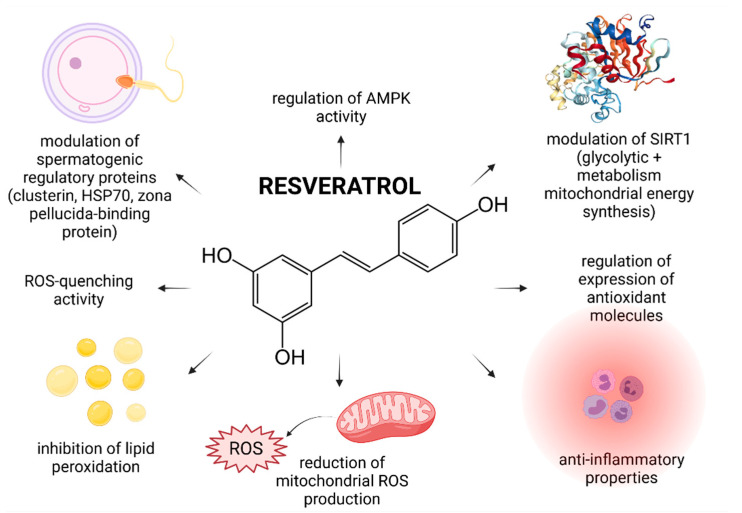
Most frequently reported beneficial effects of resveratrol on spermatogenesis, sperm structural integrity and functional activity.

In recent years, numerous studies have focused on the effects of RES on male reproductive performance and reported that RES enhances spermatogenesis by stimulating the hypothalamic-pituitary-gonadal axis, triggers penile erection, reinforces testosterone production, increases testicular sperm count and epididymal sperm motility [23,37]. Furthermore, it was suggested that RES administered in vivo or in vitro acts as an effective reactive oxygen species (ROS)-quencher and stabilizes the antioxidant balance of male reproductive cells and tissues. Moreover, first clinical studies examining the impact of RES-fortified nutraceuticals are currently underway [13]. As such, it may be hypothesized that RES presents with a complex nature and affects multiple cellular and molecular targets within the male reproductive system (Figure 1).

The first clinical trial that focused on the effects of RES on a possible improvement of male idiopathic infertility was published only recently by Illiano et al. [13]. In this prospective single-center clinical trial, patients diagnosed with oligozoospermia and/or asthenozoospermia were administered with a nutritional supplement containing 150 mg RES together with vitamins D, B6, B12 and folic acid for 6 months. The study found that while the sperm morphology, seminal pH and volume did not change during the treatment period, consumption of the supplement led to a significant improvement of the sperm concentration and motility. The authors hypothesize that this phenomenon may be primarily attributed to the ability of RES to modulate SIRT1 that controls cellular pathways crucial for glycolysis and mitochondrial energy metabolism–respiratory balance, necessary for a proper testicular function. Furthermore, it was suggested that RES stabilizes the mitochondrial genome, and aids to prevent mitochondrial DNA (mtDNA) defects, which are commonly observed in infertile patients.

While the exact role of RES in male reproduction is still not completely understood, a substantial number of animal studies indicates that the biomolecule is able to pass through the blood-testis barrier, imparting its protective effects on the testes, and subsequently on the sperm quality [37].

A solid foundation for posterior investigations of the impact of RES on male reproduction was established Juan et al. [38] according to who administration of 20 mg/kg/day RES led to an increased diameter of the seminiferous tubules, accompanied by greater sperm counts in healthy rats. Since other sperm quality parameters were not affected, the authors conclude that RES did not exhibit any toxic effects on the animals and enhanced the sperm production primarily by stimulating the hypothalamic-pituitary-gonadal axis.

Subsequently, a large quantity of reports emerged, emphasizing on ameliorative and stimulating effects of RES on the process of spermatogenesis and subsequent sperm quality that may be compromised by diseases, stress, lifestyle changes, medication, or environmental risk factors.

The effect of RES on the sperm motion and testicular oxidative profile in rats presenting with triiodothyronine-induced hyperthyroidism was investigated by Ourique et al. [39]. It was revealed that RES treatment (1 mg/kg/day or 10 mg/kg/day) counteracted a decline of sperm motility, which was accompanied by a significant prevention of lipid peroxidation (LPO) and a stabilization of catalase (CAT) and glutathione peroxidase (GPx) activities. This behavior of RES may be attributed to its exceptional antioxidant properties and ability to enhance the activity of key antioxidant enzymes. Positive effects of RES have also been studied in metabolic disorders such as diabetes. Abdelali et al. [40] demonstrated that RES ameliorated Type 1 diabetes mellitus-induced abnormal sperm formation, oxidative damage, sperm DNA fragmentation, and furthermore affected the polyADP-ribose polymerase (PARP) signaling pathway in the rat testes. The biomolecule proved to be equally effective in Type 2 diabetes, as a dose of 1.5 mg/kg RES increased the sperm count, motility and viability, and furthermore provided a significant protection to the sperm chromatin integrity and stability in streptozotocin (STZ)-nicotinamide-treated rats [41].

Possible ameliorative effects of RES on the spermatogenic dysfunction as a result of high-intensity exercise were studied by Guo et al. [42]. This study revealed that rats subjected to 9 weeks of intensive exercise and 50 mg/kg/day RES, presented with a stable sperm density. Furthermore, the concentrations of interleukin 6 (IL-6) and tumor necrosis factor α (TNF-α) were found to be significantly decreased, suggesting potent anti-inflammatory properties of RES. Using a global proteomic approach, the authors observed, that RES was able to modulate the expression levels of key of spermatogenic regulatory proteins, including Clusterin, Zona pellucida binding protein (Zpbp), Heat shock proteins and Centrin 1, indicating that RES could be indeed able to interact with molecular pathways crucial for a proper sperm production and function.

A significant impact of RES on the molecular machinery of the sperm production, maturation and vitality was reported by Shati et al. [43], who investigated possible protective roles of RES against cisplatin (Cis)-induced reproductive dysfunction in rats. It was revealed that 20 mg/kg RES administration post-Cis therapy restored all sperm parameters and prevented testicular apoptosis mediated by inhibition of key apoptotic markers, including cleaved caspase-3, p53, calpain-1/cleaved caspase-12, or p-ERK1/2, while the levels of p-Akt/p-Bad were upregulated following exposure to RES. Molecular changes in the testicular tissue were translated into a significant preservation of the sperm count, motility and morphology, a reduction of testicular apoptosis and a stabilization of the antioxidant molecules in the testes.

An inhibition of germ cell apoptosis as a result of RES supplementation was also reported in New Zealand white rabbits intoxicated with codeine. At the same time, it was observed that RES exhibited strong sperm DNA-protecting properties, as evidenced by a reduced 8-hydroxy-2’-deoxyguanosine (8-OHdG) production, which is acknowledged as a biomarker of oxidative DNA damage [44]. In the meantime, an improved histological structure of testes, accompanied by an increased sperm motility, count and viability in mice exposed to RES (2, 8 or 20 mg/kg/day) and 20 mg/kg/day morphine was reported by Jalili et al. [45]. A significant ability of RES to inhibit nitric oxidase and thus to decrease nitric oxide production was observed as well. In a later study, Jalili et al. [46] reported that RES (2, 8 or 20 mg/kg) was able to stabilize the testicular redox balance and prevent LPO in rats exposed to malathion—a highly toxic and prooxidant organophosphate.

Currently available in vitro data suggest that RES is accompanied by a peculiarity; at low doses, the biomolecule improves cell survival and vitality, while at high doses, it exhibits cytotoxic effects [47]. One of the first studies emphasizing on this dichotomy in spermatozoa was published by Collodel et al. [48]. The authors evaluated the impact of different RES concentrations (6–100 µmol/L) on swim-up human spermatozoa and noted that the best beneficial effect was reached at 6 and 15 µmol/L RES, while a complete inhibition of sperm motility was observed at 100 µmol/L RES. Similar data were observed in the case of bovine spermatozoa [49]. Interestingly, Shabani Nashtaei et al. [50] observed that while the highest RES concentrations (25 mmol/L) significantly reduced total and progressive human sperm motility, secondary kinematic parameters, such as curvilinear velocity, average path velocity and straightness were not affected. Finally, Cui et al. [51] pointed out to a significant improvement in the motion behavior and acrosin activity of spermatozoa collected from obese patients with astenozoospermia that were incubated for 30 min. in the presence of 30 µmol/L RES.

RES has been proven as an exceptional in vitro antioxidant that may be attributed to four main mechanisms of action: (a) a direct prevention of ROS overproduction in the sperm mitochondria, (b) the ability to scavenge superoxide, hydroxyl radical, and metal-induced radicals, (c) inhibition of oxidative insults to the sperm lipids, and (d) regulation of the activity of endogenous antioxidants [34,35,37,52].

Alamo et al. [53] investigated the impact of RES on human sperm damage caused by benzo-a-pyrene (BaP), a polycyclic aromatic hydrocarbon which is a potent prooxidant and promoter of caspase as well as endonuclease activation. The authors observed that 15 µmol/mL RES significantly counteracted the detrimental effects of BaP on the sperm chromatin compactness and oxidative damage to the lipids. Moreover, Tvrdá et al. [54] reported that particularly 50 µmol/L RES maintained the sperm motion and viability, stabilized the activities of antioxidant enzymes and prevented LPO in bovine spermatozoa subjected to oxidative stress induced by ferrous ascorbate.

According to Collodel et al. [40] 15 µmol/L RES preserved the chromatin texture of spermatozoa exposed to tert-butyl hydroperoxide (TBHP) which fortifies a possible utilization of RES as a supplement during cryopreservation or in vitro fertilization—intracytoplasmic sperm injection (IVF-ICSI). This potential was later confirmed by Li et al. [55] according to who the presence of 10^−4^ mol/L RES in the washing and fertilization medium significantly decreased ROS production, LPO, phosphatidylserine externalization, and protected the mitochondrial function as well as acrosomal integrity of sex-sorted bull spermatozoa, thereby increasing the blastocyst percentage and quality following IVF.

Accordingly, RES has been extensively studied as a suitable alternative supplement to cryopreservation media, primarily thanks to its ability to prevent oxidative damage to biomolecules critical for the sperm survival. An improved post thaw sperm motility, membrane stability and mitochondrial activity has been reported in human [56], bovine [57], ram [58] and boar spermatozoa [59] exposed to RES during the freezing and thawing process. Nevertheless, dose-dependent effects of RES have been frequently observed during sperm cryopreservation as well.

Branco et al. [60] reported that while 10 mmol/L RES significantly decreased DNA damage in fertile as well as infertile men, the compound was not able to prevent a post-freezing motility decline. This inability to prevent the loss of motility was observed in later studies [46,61] despite RES being effective in protecting the sperm lipids against cryodamage.

The hypothesis that RES may modulate the activity of AMP-activated protein kinase (AMPK) was postulated by Shabani Nashtaei et al. [50] who reported that 5, 15 or 25 µmol/L RES significantly increased AMPK phosphorylation, as opposed to Compound C—a known AMPK inhibitor. The exact mechanism by which this occurs however needs to be clarified further, since AMPK activation can occur through an array of mechanisms, which may include an increase in the AMP/ATP ratio, inhibition of mitochondrial ATP synthase or ROS overproduction [62,63].

Lastly, it was suggested that RES may be able to affect key paternal transcripts considered as potential markers for male fertility (protamine 1 and 2) and pregnancy success (adducin 1 alpha) in cryopreserved human spermatozoa, particularly by AMPK stimulation and improvement of interactions among mRNAs, making them more resistant to cryopreservation [64].

Summarizing the collected information, RES seems to be a complex biomolecule, able to modulate male reproductive performance at the tissue, cellular as well as molecular level. Its complexity and ability to interact with a wide array of biomolecules and signaling pathways fortify it potential as an in vivo or in vitro supplement for the prevention or management of male subfertility. Nevertheless, the existence of unclear and often contradictory data suggests that further studies are necessary to consolidate the understanding of the properties of RES and its roles in male reproduction.

## 3. Quercetin

Quercetin (3,30,40,5,7-pentahydroxylflavone; QUE) is a flavonol-type flavonoid which may be found in citrus fruits, berries, herbs and spices, red wine, cocoa, tea and fruit juices [65]. The biomolecule has been reported to exhibit anti-inflammatory, anticarcinogenic, antibacterial, anti-aggregatory and antidiabetic effects [65,66]. Within the flavonoids, QUE is considered to be the most potent scavenger of ROS and nitric oxide [67]. These antioxidant properties could be attributed to its direct ROS and reactive nitrogen species (RNS)-trapping properties, ability to chelate metals, and to interact with lipid bilayers, modulate enzymes and/or induce repair mechanisms. Moreover, QUE has been reported to substantially fortify the endogenous antioxidant defense mechanisms because of its contribution to the total antioxidant capacity. Additionally, QUE may be effective because of its ability to interact with and penetrate through lipid bilayers [68].

**Figure 2:**
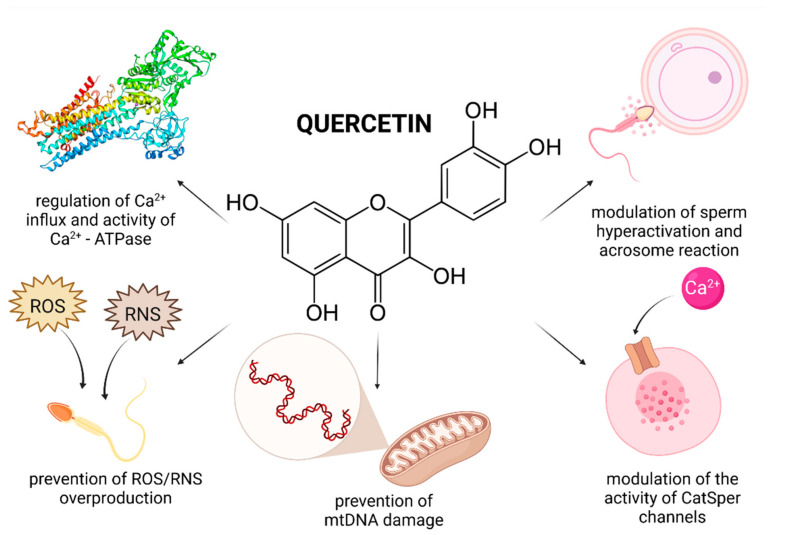
Most frequently reported beneficial effects of quercetin on spermatogenesis, sperm structural integrity and functional activity.

Evidence on the impact of QUE on male reproduction is still controversial and inconsistent. While most studies emphasize on stimulating and protective effects of QUE on male reproductive performance, there is still a solid number of reports which indicate its potential toxic or adverse effects on reproductive cells and tissues. This controversy is primarily caused by a lack of understanding on the exact bioavailability and behavior of QUE in living tissues. Furthermore, as opposed to RES or lycopene, human trials have not been taken up yet. By and large, in vivo animal studies indicate beneficial effects of QUE on the testicular function (Figure 2).

The effects of QUE on the testicular structure and semen quality depending on its dose and time of administration have been studied by Taepongsorat et al. [69]. Male rats were injected with 0, 30, 90 or 270 mg/kg b.w./day QUE and its impact was assessed following 3, 7 and 14 days of treatment. The shortest time of QUE treatment had no effects neither on the weights of the reproductive organs nor on the sperm quality. Nevertheless, a significantly increased testicular and epididymal weight, accompanied by an increased sperm motility, viability and concentration was observed following administration of 90, or 270 mg/kg b.w./day QUE for 7 and 14 days, respectively. The authors speculate that QUE may affect the sperm quality indirectly, either through a stimulation of other sex organs or via an enhancement of the hypothalamic-pituitary-testicular axis. A direct effect on the sperm quality was not proposed, as the observed changes might have been attributed to sperm retention in the epididymis and epididymal lumen dilatation, leading to an increased quality of the stored sperm reserve.

According to Yelumalai et al. [70] STZ-nicotinamide induced male diabetic rats treated orally with QUE (10, 25 and 50 mg/kg/b.w.) presented with an increased sperm count and motility, viability and membrane integrity while the number of spermatozoa with abnormal morphology decreased. Furthermore, the activities and mRNA expression levels of major antioxidant enzymes (superoxide dismutase—SOD, CAT, GPx) increased while LPO, NF-κβ and TNF-α levels decreased, suggesting strong antioxidant and anti-inflammatory properties of QUE in counteracting reproductive complications resulting from diabetes.

Furthermore, it was reported that QUE (30 mg/kg) administered orally improved the epididymal weight and testicular length, stabilized the architecture of seminiferous tubules and decreased the incidence of testicular apoptosis in rabbits subjected to induced heat stress. A significant improvement was also observed in the epididymal sperm motility and kinetics, viability, mitochondrial activity, and acrosome integrity. It was postulated that QUE affects the sperm and testicular configuration while minimizing oxidative insults, which in turn protects the testes and spermatozoa against heat stress-induced damage [71].

QUE proved to be a highly effective biomolecule in toxicological studies as well. Jahan et al. [72] observed that in rats intoxicated with arsenic QUE treatment (50 mg/kg) significantly prevented tissue deposition of the heavy metal within the testis, as revealed by an improved testicular structure and a significantly higher daily sperm production. Sperm DNA damage, induced by arsenic, was also significantly reversed. Furthermore, Ben Abdallah et al. [73] reported that administration of 50 mg/kg/day QUE along with lambda-cyhalothrin (LTC) significantly prevented a decrease in functional sperm parameters and LPO products while increasing the testicular activities of antioxidant enzymes as well as glutathione (GSH) concentration. Bu et al. [74] noted that QUE supplementation (100 mg/kg) significantly restored the depletion level of GSH and the activities of SOD and GPx in mouse germ cells intoxicated with 3-methyl-4-nitrophenol from diesel exhaust particles.

As discussed earlier, we must acknowledge that in case of a broader concentration range, QUE may act dose dependently as either a stimulant at low doses or as an inhibitor at high doses [68]. The flagship study on this issue was published by Ranawat [75] who assessed the effect of intraperitoneal QUE administration (2, 8 and 20 mg/kg) on male reproductive function in adult mouse for two weeks. QUE increased the generation of ROS and LPO in the testes with a concomitant decrease of the sperm count and motility in a dose dependent manner. Activities of SOD and CAT as well as the levels of GSH were found to be decreased in a dose dependent manner. Testicular histomorphology was also altered, and a significant loss of various germ cell populations was observed depending on the dose of QUE applied. This discrepancy was fortified by Aravindakshan et al. [76] who showed that administration of a higher QUE dose (300 mg/kg b.w.) reduced the fertility rate of male rats during the first two matings, however the reproductive function was recovered several weeks post-treatment.

This controversy may be explained by the fact that QUE may undergo a process of oxidation, while its oxidized form will arylate GSH and protein thiol groups, ultimately leading to its consumption. Low GSH concentrations will subsequently increase cytosolic calcium (Ca^2+^) concentration and leakage of lactate dehydrogenase (LDH) [77]. If the cytosolic free calcium levels trespass their physiological limits, excessive Ca^2+^ may either cause cytotoxicity or trigger programmed cell death [78]. This sequence of intracellular events will ultimately lead to an inhibition of sperm motility and germ cell depletion even though initially QUE acts as an antioxidant. As such, doses of the biomolecule must be considered carefully, in order not to trespass the delicate threshold between its beneficial and toxic effects on the male reproductive system.

In the meantime, most in vitro studies on QUE as an alternative supplement for sperm processing, culture and preservation indicate that the biomolecule has a stimulating effect on the structural integrity and functional activity of the male gamete. 

According to Diao et al. [79], 10 μmol/L QUE could significantly improve the motion behavior of spermatozoa collected from leukocytospermic patients. In vitro studies on animal spermatozoa also agree that exposure of male gametes to particularly QUE concentrations ranging between 10 and 25 μmol/L may lead to a higher preservation of motility in bovine [80] and boar spermatozoa [81,82]. Furthermore, pivotal experiments on cryopreserved boar [83], stallion [84], red fowl [85] and ram [86] spermatozoa suggest that low QUE concentrations administered to the freezing and thawing medium improve the sperm motility and a subsequent fertilization potential.

Furthermore, El-Khawagah et al. [87] observed that besides overall motility, 10 μmol/L QUE positively affected secondary kinematic parameters of buffalo bull spermatozoa, including their progressive motility, curvilinear velocity (VCL) and amplitude of lateral head displacement (ALH). Similar effects of QUE on the sperm velocity parameters were reported in studies on frozen-thawed stallion [84] and boar [83] spermatozoa, and indicated a positive correlation between QUE supplementation, sperm fertilizing capacity, in vitro fertilization success and pregnancy rates.

Several explanations are offered to an increased sperm motion behavior following in vitro QUE administration. It was previously suggested that QUE could modulate the activity of CatSper channels which mediate progesterone induced Ca^2+^ influx and play a role in the sperm hyperactivation and acrosome reaction [88]. Furthermore, it may be feasible to assume that the antioxidant properties of QUE enable the molecule to further stabilize progesterone receptors on the sperm membrane [79]. Beneficial effects of QUE on the sperm motion may be furthermore related to its interaction with Ca^2+^-ATPase, a key enzyme involved in supporting sperm motility, possibly by its role in intracellular cyclic adenosine monophosphate (cAMP) production [89].

Recent in vitro studies indicate that QUE is a highly efficient biomolecule in preventing the loss of the mitochondrial membrane potential [81] and a subsequent mitochondrial dysfunction by its ability to accumulate inside the mitochondria [90] and to control ROS production by its antioxidant activity [87]. The mitochondrial system is considered to be the primary source of intracellular ROS, whereas QUE may play important roles in the absorption and neutralization of ROS created mainly by the activity of NADPH oxidase and NADH-dependent oxidoreductase, localized in the inner mitochondrial membranes [65,66,91]. Furthermore, it was revealed that QUE treatment led to a higher stabilization of the mitochondrial genome as evidenced by a significantly decreased sperm mtDNA copy number in leukocytospermic semen samples [79]. Correspondingly, the levels of cytochrome B, NADH 5 [79] and succinate dehydrogenase were significantly increased after treatment with QUE, suggesting a protective effect of the biomolecule on important components of the mitochondrial respiratory chain.

Protective effects of QUE on the mitochondrial structure and function, coupled with its straight-forward antioxidant properties could furthermore decrease possible ROS leakage from the sperm mitochondria to the nucleus, minimizing the susceptibility of the male genome to oxidative insults. Correspondingly, a significantly reduced DNA fragmentation following in vitro exposure to QUE was found in bovine [92], stallion [93] and boar spermatozoa [81]. Moreover, it was suggested that 30 µmol/L QUE could prevent chromatin distortion caused by the exposure of human spermatozoa to tert-butylhydroperoxide (TBHP) [94].

The ability of QUE to prevent or counteract ROS overproduction has been acknowledged in numerous reports. According to Tvrda et al. [80,81,92] QUE was highly efficient in opposing high levels of superoxide, which is considered to be the prevalent ROS produced by the sperm cell, and the first one to initiate the Fenton reaction. Further reports on the effects of QUE on human [94], bovine [14] and rat [42] spermatozoa speculate that QUE could exhibit its superoxide trapping properties through the inhibition of NADPH oxidase and/or NADH-dependent oxidoreductase; SOD mimicking; or direct superoxide quenching. Hence, it may be suggested that the biomolecule may be particularly efficient during the initiation of oxidative chain reactions, keeping superoxide in physiological levels.

This property of QUE also enables the biomolecule to prevent further increase of hydrogen peroxide (H_2_O_2_) production, which may result in LPO. As such, a significantly decreased concentration of malondialdehyde (MDA) in spermatozoa exposed to QUE is a frequently observed phenomenon, as reported in humans [94], goats [95], bulls [80,87], boars [81], and stallions [96].

Despite a convincing body of evidence on the protective effects of QUE on male gametes, the quercetin paradox has been often observed in vitro as well, particularly in cases when high doses of QUE were supplemented [68,77].

An in vitro study carried out on human semen samples showed that treatment with 50–100 mmol/L QUE resulted in an irreversible and dose-dependent sperm motility inhibition [97]. A disruption of sperm motion and viability was observed in the case of bull [80,92] and boar [81] spermatozoa as well. A decreased total and progressive sperm motility, velocity, wobble, oscillation index and a lower percentage of fast cells were reported by Silva et al. [98] and Borges et al. [99] who studied the effects of QUE on frozen goat spermatozoa.

Adverse effects of QUE on the sperm motion behavior may be directly associated with the previously discussed ability of the biomolecule to modulate Ca^2+^-ATPase activity [100]. Inappropriately high doses of QUE may decrease the activity of the enzyme, which will subsequently lead to an accumulation of Ca^2+^ in the cell. Supraphysiological Ca^2+^ levels will then block the motion apparatus of the sperm cell, lowering the cAMP concentration and restricting the ATP supply with a concomitant fall in its motility [87,100]. High intracellular Ca^2+^ concentrations may also decrease the level of tyrosine phosphorylation events that are absolutely required for the maintenance of acrosome reaction and capacitation [100]. In addition, QUE exhibited significant inhibitory effects on the hyaluronidase activity and sperm penetration ability in non-capacitated, capacitated and acrosome-reacted cynomolgus monkey sperm in a dose-dependent manner [101].

As such, conflicting biological effects of QUE should be carefully considered before the biomolecule is supplemented to extended spermatozoa. Furthermore, the toxicity of QUE needs to be monitored closely, since its “double-edged sword” behavior is dependent on its dose, species studied in the experiment as well as experimental settings.

## 4. Lycopene

Lycopene (ψ,ψ-Carotene) (LYC) is a natural carotenoid, high concentrations of which may be found in ripe tomatoes, watermelons, apricots, pink grapefruits, or papaya [102]. Interestingly enough, the bioavailability of LYC from fresh foodstuffs is relatively low, however this obstacle may be trespassed by heat, processing or co-delivery of fruits and vegetables with foods that contain fat [103].

Although used as a food colorant for decades, LYC has only recently emerged as a subject of scientific interest with respect to its biological activity. LYC is a very powerful antioxidant, which has been shown to quench singlet oxygen twice as effectively as β-carotene and ten times faster when compared to α-tocopherol [102,103].

Numerous reports have shown that this red pigment exhibits protective effects against malignant, cardiovascular, neurodegenerative and eye diseases [102]. It has been reported that LYC accumulates in lipid-rich seminal prostasomes, providing them protection against degradation [104], which is why the molecule is particularly effective in the prevention of prostate cancer [105]. Furthermore, it has been revealed that LYC concentrations in the testes are higher in comparison to other organs indicating that the molecule is likely to play an important physiological role in the process of spermatogenesis [25]. This hypothesis was further fortified by Palan et al. [106] who reported that the concentration of LYC was significantly lower in the seminal plasma collected from infertile men. Additional research [107,108,109] has revealed that the amount of LYC in the seminal plasma increases with oral supplementation of the compound. Summarizing the above-mentioned information, it may be strongly assumed that the intake of LYC will offer a higher level of protection against testicular oxidative stress, and hence possible male reproductive dysfunction.

**Figure 3:**
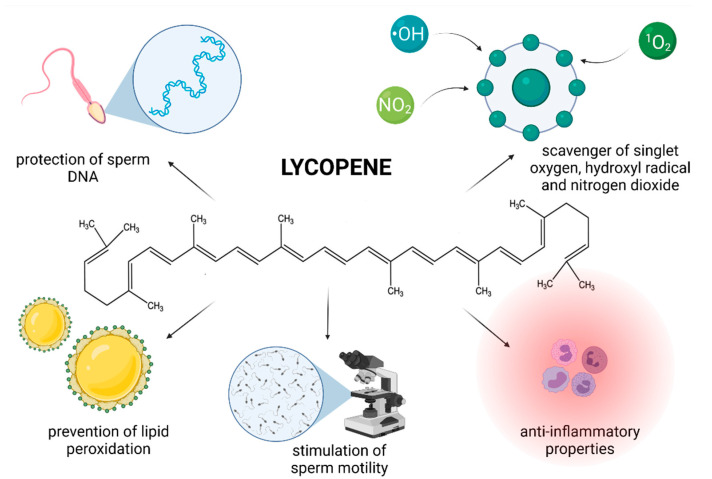
Most frequently reported beneficial effects of lycopene on spermatogenesis, sperm structural integrity and functional activity.

Although the exact mechanism of action by which LYC exerts its biological activity is still poorly understood, recent studies provide evidence that LYC may be useful in the prevention or management of male infertility (Figure 3).

Williams et al. [108] studied the effects of a 12-week supplementation with 14 mg/day of lactolycopene (equivalent to the consumption of 2 kg of cooked tomatoes or 2 tablespoons of concentrated tomato puree) on standard sperm quality parameters of healthy volunteers with no previous fertility issues. Whilst the sperm concentration or DNA integrity were not affected, a significantly improved proportion of fast-progressive sperm as well as a higher amount of morphologically intact male gametes were observed at the end of the experiment, which may be eventually welcomed by infertile men. Indeed, a 12-week LYC supplementation resulted in a significant improvement of the sperm count and concentration in infertile men, however no changes were observed in the sperm morphology [110]. In Mohanty’s et al. study [111], 50 oligoasthenozoospermic males consuming 8 mg LYC over an extended period showed a significant increase in the sperm count and concentration, while according to Gupta and Kumar [112], 30 idiopathic infertile men presented with a 53% increase in sperm concentration following consumption of 2 mg/day LYC for 3 months.

In their study, Yamamoto et al. [109] revealed that infertile patients who consumed tomato juice (containing 30 mg LYC) for 12 weeks, presented with a significantly decreased the concentration of seminal leukocytes suggesting a lower risk for inflammation and sperm damage because of leukocytospermia. Furthermore, the motility rates were significantly higher in the experimental group in comparison with the control indicating that commercially available food, such as tomato juice, might be beneficial to counteract male infertility.

Several clinical trials also focused on the antioxidant properties of LYC in the context of oxidative stress-compromised male reproductive function. Filipcikova et al. [113] observed a significant decrease of MDA production in the seminal plasma of infertile men consuming 10 mg of highly purified LYC from tomato puree and emphasized on the efficacy of LYC in stabilizing the levels of polyunsaturated fatty acids in the sperm membranes. Taş et al. [114] speculated that this LPO-preventing potential lies in the ability of LYC to become entrapped in the hydrophobic core of membranous constituents in spermatozoa. Moreover, LPO was reportedly reduced by consuming tomato juice fortified with vitamin C for 2 weeks [115], where the authors putatively speculated that vitamin C might have acted in a synergy with LYC present in the tomato juice. Interestingly, although a decrease of MDA and a concomitant increase in the GPx activity were observed by Nouri et al. [110], these changes were not statistically significant. Nevertheless, the authors conclude that LYC supplementation may play a role in the improvement in the established functional criteria of fertility, by stabilizing the oxidative balance in semen which may be subsequently translated into improved sperm parameters.

Different mechanisms of action of LYC have been proposed, however the most prominent seems to be mediated via its ability to prevent ROS overproduction. LYC is known to be a highly efficient scavenger of singlet oxygen, hydroxyl radical and nitrogen dioxide [116,117]. As such, the compound may offer protection against oxidation of biomacromolecules crucial for a proper reproductive function [108,110,114]. Moreover, since LYC is lipophilic and frequently found in cell membranes [102,103] it is likely to be present in sufficient amounts to protect critical sperm structures against oxidative insults. Other mechanisms that have been suggested include that of indirectly increasing the amount and/or activity of antioxidant enzymes [25,117] and decreasing the transcription of proinflammatory factors [18,118].

With respect to animal studies, while Mangiagalli et al. [119] reported that administration of 0.1 or 0.5 g/L LYC had no significant impact on the motility rate or forward progressive motility of spermatozoa collected from healthy rabbits, an array of toxicological studies emphasizes on a protective effect that LYC exerts in the male reproductive system compromised by irradiation [120] or drugs [121,122].

According to Dobrzyńska and Gajowik [120] irradiated mice that were exposed to lycopene (0.15 and 0.30 mg/kg) presented with an increased sperm count and a lower percentage of abnormal spermatozoa and/or spermatozoa with a fragmented DNA molecule, suggesting that LYC can ameliorate the harmful effects of irradiation on male gametes.

Türk et al. [121] indicated that LYC administration in rats treated with cyclosporine A (CsA) significantly increased the sperm concentration, motility, and decreased ROS generation in comparison to the CsA-treated control, confirming the role of LYC as a potential protective agent against structural and functional damage as a result of ROS-inflicted testicular damage. A significant antioxidant activity of LYC was furthermore reported by Ateşşahin et al. [122] according to who the presence of LYC significantly improved the semen quality and testicular antioxidant capacity of rats treated with cisplatin. According to Aly et al. [123], LYC supplemented before lipopolysaccharide treatment attenuated the mitochondrial damage in male germ cells. This effect may be attributed to the lipophilic nature of the LYC, which enables the biomolecule to accumulate in the membranous structures and lipoproteins, and subsequently stabilize the mitochondrial metabolism [25]. Protective effects of LYC were accompanied by a decrease of MDA and H_2_O_2_ generation, suggesting a ROS-trapping ability of this carotene. Moreover, LYC treatment prevented the testicular decrease of SOD, CAT, GPx and glutathione reductase (GR) activities, normalized GSH and vitamin C concentrations which subsequently contributed to the ROS-scavenging ability of the reproductive system. A stabilization of the testicular oxidative profile alongside an improved activity of SOD, CAT, GPx and GSH followed by a decrease of H_2_O_2_ production and MDA synthesis in male reproductive cells and tissues following exposure to bisphenol A [124], cyclosporine A [121] and cisplatin [125] were reported as well.

At the same time, current evidence points out to the potential of LYC as a suitable antioxidant supplement to preservation media for fowl [126], ram [127] and bovine semen exposed to low temperatures [128,129]. All studies agree that LYC supplementation prevented a decline in the sperm motility and prevented the occurrence of sperm abnormalities, acrosome damage or dead sperm, most likely as a result of specific protective effects of this molecule against cell damage through its ROS-quenching abilities and prevention of LPO.

In contrast, Zini et al. [130] observed that pre-incubating human sperm with LYC did not reverse the loss of motility caused by subsequent addition of H_2_O_2_ to the culture. Failure of LYC to stabilize sperm motility in this case could be attributed to the presence of high concentrations of H_2_O_2_, a well-known lipid soluble ROS which may be able to quickly escape the ROS-trapping abilities of LYC and subsequently curb the sperm activity through numerous oxidative mechanisms. Nevertheless, sperm preincubation with LYC caused a significantly lower DNA damage which may be useful particularly in cases where ICSI is applied, which does depend primarily on the sperm DNA stability rather than its motility [131]. Furthermore Rosato et al. [132] showed that semen extenders containing LYC provided a higher protection to the DNA molecule when turkey semen samples were refrigerated or cryopreserved. Taken together, these studies imply that LYC is able to prevent DNA damage in spermatozoa, therefore increasing the chances of successful fertilization of the oocyte and embryogenesis. This hypothesis is in agreement with previous reports on mammalian germ cells in which the extent of damage to the sperm DNA was assessed using the Comet assay, acridine orange test, the sperm chromatin structure assay or the quantification of 8-OHdG, a direct marker of oxidative damage to DNA [128,129,130,132].

Numerous earlier studies have reported a significant decrease in LPO following LYC supplementation [117,126,129,132]. Nevertheless, Zribi et al. [133] indicated that although oxidative stress was linked to DNA damage, no correlations were recorded between ROS production and MDA amounts. Moreover, several authors have suggested that although LYC is an antioxidant, it failed to reduce the extent of LPO in bovine [134] and ram [135] sperm following cryopreservation. Due to contradictory data on the potential of LYC in preventing peroxidative damage to lipids, it may be hypothesized that LYC may not play a leading role in counteracting LPO during sperm cryopreservation in comparison to other antioxidant supplements traditionally added to the semen extenders [129].

Summarizing the collected data, two hypotheses may emerge to explain positive effects of LYC on either improving or restoring male fertility. One suggests that LYC is a lipophilic substance which easily passes through the cell membranes and quickly enters the cell where it stabilizes and fortifies the inherent antioxidant network. Another one assumes that LYC binds to the membranous structures where it plays an important role in the protection of lipoproteins against oxidative insults. It is plausible to assume that the provitamin A activity of β-carotene has a direct effective role in this protective mechanism of action [136].

## 5. Catechins

Catechins are polyphenolic flavonol compounds which are regarded to be the key bioactive components of green tea. Furthermore, these may be found in black grapes, strawberries, and apricots [137]. Green tea catechins are by and large represented by four biomolecules, specifically epicatechin (EC), epigallocatechin (EGC), epicatechin-3-gallate (ECG) and epigallocatechin-3-gallate (EGCG) [26]. Catechins have been reported to possess a wide spectrum of biological activities and may be useful in the prevention of cardiovascular diseases, cancer, osteoarthritis, and Parkinson’s disease [137,138]. Furthermore, the biomolecules are able to modulate the metabolism of carbohydrates and lipids, thus acting in the prevention of diabetes mellitus Type 2 and liver diseases [138].

Catechin polyphenols are known to possess highly efficient ROS-scavenging activity which has been estimated to be 20 times higher in comparison to vitamin C [139]. These biomolecules are effective chelators of transition metals including cadmium and chromium. Furthermore, the ability of catechins to reduce iron and copper enables them to act in the prevention of the Fenton reaction and a subsequent production of the highly reactive hydroxyl radical [103,140].

In addition, catechins are reported to modulate the levels of monoamines, which control sexual and reproductive behavior. According to Rai et al. [141], 50 mg/kg (+)-catechin was found to enhance sexual behavior in rats, while being safe on the histology of testes, sperm count, motility, and morphology parameters.

Animal models have been used in several in vivo studies to assess the impact of catechins in improving sperm quality, which may provide valuable information for their future potential in the management of male infertility.

According to Ding et al. [142] EGCG administration (50 mg/kg) to mice subjected to intermittent irradiation provided protection against short-term germ cell loss and mitigated radiation-inflicted testicular OS. Furthermore, it was proposed that the biomolecule stimulates and protects spermatogenic recovery and prevents germ cells from radiation-induced apoptosis, leading to a higher sperm production, motility, and a decreased frequency of oxidative insults to the process of spermatogenesis. Awoniyi et al. [143] reported that supplementation of green tea extracts rich in catechins significantly improved the sperm count and motion characteristics of spermatozoa collected from rats treated with TBHP. Furthermore, the authors observed that catechin administration resulted in an improved oxidative profile, stabilization of endogenous antioxidants and prevention of an excessive production of lipid peroxides in epididymal sperm.

In the meantime, Zanchi et al. [144] evaluated the potential of green tea infusion (250 mg/kg) in alleviating cyclophosphamide-induced testicular toxicity in mice. In this study, green tea catechins prevented excessive lipid peroxidation, protein carbonylation, DNA fragmentation, while at the same time, a restored glutathione peroxidase and S transferase activity was observed in mice testes. This stabilization of the oxidative balance was translated into an increased sperm concentration in the epididymis. Green tea catechins were also reported to provide protection against testicular damage induced by doxorubicin. Mice administered with 200 mg/kg green tea presented with a significantly higher sperm density and motility, alongside an improved histology of the testicular tissue [145]. Green tea polyphenols also showed to be effective in the prevention of reproductive failure caused by hyperthermia on semen parameters. Rats subjected to scrotal heat stress which received 500 or 750 mg/kg green tea extracts, presented with a significantly restored sperm concentration, motility, and membrane integrity [146].

**Figure 4:**
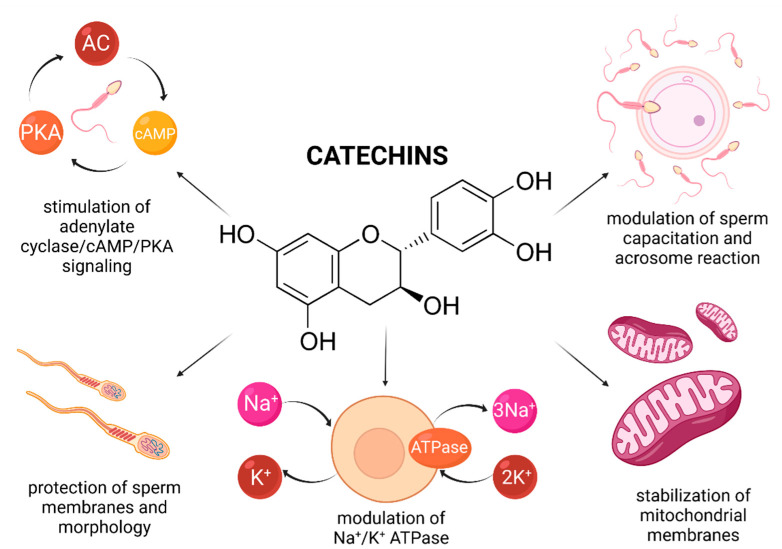
Most frequently reported beneficial effects of catechins on spermatogenesis, sperm structural integrity and functional activity.

Recently, a variety of studies has emerged indicating a potential role of catechin supplementation to sperm storage and/or cryopreservation media. Furthermore, it has been reported that catechins may confer in vitro protection against oxidative insults to male gametes (Figure 4).

In vitro animal studies revealed that catechin concentrations oscillating between 25–100 μmol/L led to a higher motility and membrane integrity preservation of extended and/or cryopreserved bull [147,148], canine [149], caprine [150,151] and boar spermatozoa [152]. This may be explained by a high ability of catechins to be incorporated into the plasma membrane, and thus avoid possible fluctuations in ion concentrations and alterations of the Na^+^/K^+^ ATPase, which may have lethal consequences to the sperm survival [153]. Consequently, the stabilization of membrane fluidity resulted in a sperm cell that was better equipped to withstand a variety of physical and environmental assaults [150].

In case of human spermatozoa, it was revealed that lower doses of EGCG (ranging from 2 to 20 μmol/L) affected the estrogen receptor of male gametes, and subsequently increased the cholesterol efflux and tyrosine phosphorylation which manifested itself in an increased sperm motility, viability, and phosphorylation of proteins affecting cell survival (Bcl2, Akt, and Src) [154]. Furthermore, administration of 2 and 20 μmol/L EGCG to the semen extender stimulated the adenylate cyclase/cAMP/PKA signaling, which, alongside protein phosphorylation, plays key roles in the process of sperm capacitation and acrosome reaction [155]. A more detailed molecular analysis revealed that the presence EGCG was associated with a reduction of triglycerides, induction of lipase and G6PDH activity, indicating an increased energy expenditure of the sperm cell. These findings were later confirmed by Spinaci et al. [156] who observed that EGCG treatment increased the number of porcine spermatozoa able to actively bind to the zona pellucida of the oocyte. As such, it may be concluded that catechins may be capable of exerting some impact on the sperm cell, resulting in a modulation of capacitation and acrosome reaction, even under ex vivo conditions.

Enrichment of semen extenders with tea extracts rich in catechins has been reported to lead to an increase in the overall sperm viability and mitochondrial activity [148,149,150]. A possible explanation for the beneficial effects of catechins on the sperm mitochondrial metabolism was offered by Uekusa et al. [157] who took advantage of nuclear magnetic resonance spectroscopy to reveal a high affinity of catechins for the sperm mitochondrial membranous structures. A proper stabilization of the mitochondrial integrity, alongside prevention of ROS overgeneration in the presence of catechins may ensure a more effective function of the sperm mitochondrial compartment. Furthermore, Nagata et al. [158] reported that a synergism exists between catechin and glutathione peroxidase, that may contribute to a more effective stabilization of the intracellular antioxidant system of the male gamete [148].

Despite the beneficial effects of catechins on male fertility as mentioned above, their possible negative effects on male reproductive organs and cells have been reported by other authors. Relatively high doses of catechins have been shown to cause inhibition of spermatogenesis alongside alterations to the morphology and activity of spermatozoa [159].

According to Jamalan et al. [160], high concentrations of catechins (25–1000 μmol/L) did not exhibit any significant protective effect on human spermatozoa exposed to cadmium, aluminum, or lead. What is more, Moretti et al. [94] observed that 200 μmol/L and 400 μmol/L EPI had a compromising effect on human sperm motion behavior, overall viability, or the extent of LPO when male gametes were subjected to TBHP. A similar observation was reported by Silva et al. [151] who observed that the treatments with (+)-catechin or EGCG, at higher concentrations (50 to 100 µmol/L), had an inhibitory effect on goat sperm kinematics.

A possible explanation for this phenomenon may lie in the ability of high doses of catechins to inhibit F0F1-ATPase and cyclooxygenase (COX) which contribute to ATP synthesis. Furthermore, COX modulates the production of prostaglandins, which affect sperm motility [62,161]. Finally, it has been postulated that elevated concentrations of catechins may exhibit antiestrogenic properties [154]. In this sense, catechins may be defined as natural dose-dependent motility inhibitors. As such, it must be emphasized that the dosage may be the single most important factor deciding whether catechins will exhibit beneficial or detrimental effects on the sperm function.

Another aspect that must be taken into consideration is an increasing evidence on “double-edged sword” properties of catechins, as these may be converted to pro-oxidant derivatives [162]. Catechins are relatively unstable and can contribute to oxidative tension by undergoing autooxidation. According to Sang et al. [163] the stability of catechins directly depends on their concentration, incubation temperature, pH of the semen extender or the presence of oxygen. As such, all these variables must be considered before catechins may be used as supplements for sperm processing and preservation.

## 6. Curcumin

Curcumin [1,7-bis(4-hydroxy-3-methoxyphenyl)-1,6-heptadiene-3,5-dione] (CUR) is the principal curcuminoid of the dietary condiment and herbal remedy turmeric. This yellow spice is obtained by crushing the roots of the plant *Curcuma longa* Linn. into powder. CUR is responsible for the color of turmeric and is nowadays recognized to be responsible for most of its therapeutic effects [21]. Numerous cell culture and animal studies suggest that curcuminoids exhibit neuroprotective, anti-inflammatory, antitumor and radioprotective activities [164]. Preliminary clinical trials also suggest a potential therapeutic role of CUR in the management of a variety of malignant and chronic inflammatory diseases. CUR is an active scavenger of superoxide and hydroxyl radicals as well as nitrogen dioxide. The biomolecule is also a potent LPO inhibitor [164,165]. Contradictory evidence is, however, available on the effects of CUR on male reproductive function.

**Figure 5:**
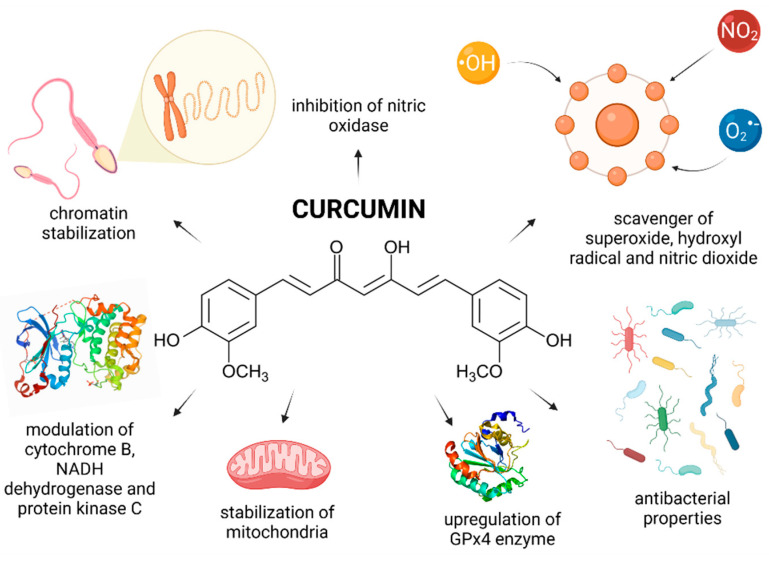
Most frequently reported beneficial effects of curcumin on spermatogenesis, sperm structural integrity and functional activity.

Most in vivo reports provide evidence for beneficial and protective roles of CUR on the testicular physiology, spermatogenesis, and sperm physiology (Figure 5). As reported by Kazemizadeh et al. [166] semen concentration, total sperm production, progressive motility and membrane integrity were improved correspondingly to the increasing CUR doses (10, 20 or 30 mg/bird) in broiler roosters, which manifested itself in a significantly higher sperm-egg penetration membrane and fertility rates. A similar observation was recorded by Iranloye and Uweru [167] who indicated that CUR (50, 100 or 150 mg/kg) enhanced fertility in male Wistar rats as evidenced by an increased sperm concentration, motility, and morphology with a concomitant boost of spermatogenic activity in the seminiferous tubules.

Furthermore, it has been reported on numerous occasions that CUR presents with the ability to reverse reproductive toxicity caused by a variety of endogenous or exogenous triggers. According to Izadpanah et al. [168] 100 mg/kg CUR had ameliorative effects on varicocelized rats, as confirmed by a significant restoration of the sperm count, motility, and morphology. Higher testicular biopsy score values, increased seminiferous tubules diameter and testicular weight alongside improved sperm parameters such as count, motility and morphology were furthermore observed following CUR supplementation to experimental animals exposed to di(2-ethylhexyl)phthalate [169], metronidazole [170], chromium [171], lindane [172], cyclophosphamide [173], morphine [174], fluorescent radiation [175] or protein deficiency, indicating a versatility of the biomolecule that may be applied under different circumstances.

An important mechanism of action of CUR has been suggested to lie in its ability to competitively inhibit the cytochrome P450 isoenzymes in charge for the metabolic activation of numerous carcinogens [176]. CUR is also able to protect the testicular tissue by suppressing NF-κB DNA-binding activity and, thus affect the expression patterns of genes that require NF-κB for their activation [177]. This in turn may significantly contribute to the reduction of apoptosis during spermatogenesis. Shahedi et al. [178] found that 100 mg/kg CUR had a beneficial effect on the cell survival and sperm nuclear condensation in mice subjected to testicular torsion, particularly during an extended period of treatment. An important observation raised by the authors was that CUR exhibited notable chromatin-stabilizing properties driven by its protective effects on the lysine-rich histones, chromatin condensation, cytosine-rich and guanosine sequences which contribute to the degree of chromatin protamination in spermatozoa.

Most animal studies agree on significant antioxidant properties of CUR. The biomolecule presents with the ability to prevent the production of superoxide and hydroxyl radicals by ceasing the oxidation of iron through the Fenton reaction. However, another reason for the improvement of male reproductive performance may lie in a stabilization of intrinsic ROS-scavenging molecules because of CUR administration [179]. Furthermore, it was suggested that CUR may inhibit nitric oxidase activity, which leads to decreased nitric oxide levels in the testicular tissue, and hence in the prevention of the oxidative overload which may compromise testicular function during varicocele [168]. Zha et al. [180] reported that CUR reduced testicular apoptosis caused by diabetes by affecting apoptotic proteins, decreasing NADPH activity, and restoring enzymatic antioxidants. Furthermore Wei et al. [181] indicated that CUR had the ability to diminish xanthine oxidase activity during testicular torsion-detorsion. Finally, it has been reported on numerous occasions that CUR prevents oxidative insults to lipids present in the reproductive cells and tissues [170,171,172,173,174,175,176,177,178]. As such, it can be assumed that the antioxidant nature of CUR enables the biomolecule to modulate the oxidative balance directly or indirectly, and thereby reduce adverse effects of testicular stress and mitigate sperm damage.

Despite a promising nature of CUR in the management of testicular toxicity, there are still numerous challenges that limit its practical use. CUR has a low aqueous solubility, is sensitive to light and relatively unstable for an extended period, resulting in poor CUR bioavailability [20]. As such, a variety of studies employed encapsulation and/or nanoparticles for CUR, that may enhance its in vivo bioavailability and maximize its therapeutic potential [182,183]. According to Ahmed-Farid et al. [184], lower concentrations of encapsulated CUR proved to be equally effective as traditionally administered CUR in preventing apoptosis of male reproductive cells and in stabilizing the testicular structure and function. A significant reduction of the testicular oxidative stress and a decrease of sperm abnormalities following encapsulated CUR administration was recorded as well.

Nevertheless, Xia et al. [185] indicate a negative involvement of CUR in spermatogenesis as their study revealed an inhibiting effect of CUR on the growth of mouse spermatids, accompanied by an increased apoptosis. More importantly, CUR acted as a histone acetylase inhibitor, leading to an inhibition of transcription in the spermatids and a disrupted dynamics of chromatin-associated factors considered to be a vital reprogramming mechanism during spermiogenesis. As such, a cautioned and more complex investigation of potential toxic and contraceptive effects of the biomolecule are still relevant. Furthermore, it has been emphasized that the reproductive toxicity of nano-formulated CUR needs to be prudently evaluated before its application, as the nanoparticles per se could be harmful to the differentiation of spermatogonia, elongation of spermatids, as well as the motility of mature spermatozoa [186].

In vitro processing and storage of semen leads to additional mechanical, thermal, osmotic, and oxidative stress inflicted on male gametes, which builds up a foundation for studies investigating the potential of CUR as an alternative supplement to semen extenders and cryopreservation media. According to an array on studies, CUR acted as an effective promoter of sperm motility in extended or cryopreserved rat [187], boar [188], bull [189], ram [190], rabbit [191], and human semen [192]. Furthermore, it was observed that CUR supplementation to semen extenders may lead to a lower percentage of alterations to the membrane integrity and acrosome stability [189,190,193,194] while the molecule exhibits a significant protection to the sperm mitochondrial activity, and thus enables a prolongation of the sperm vitality during liquid storage or cryopreservation [187,189,190]. CUR also exhibited its antibacterial activity as it was able to maintain a satisfactory rabbit sperm structural integrity and functional activity under in vitro induced bacteriospermia caused by *Enterococcus faecalis* [195].

Numerous reports suggest that one of the possible mechanisms by which CUR exerts a protective role on the sperm structure or function is through its ability to scavenge ROS directly, or to stimulate the internal ROS-quenching and detoxification system of male gametes under induced oxidative stress [196], which is intricately related to male fertility. A significant ex vivo preservation of sperm DNA integrity was recorded following CUR administration, most likely because of its antioxidant properties. Furthermore, CUR mitigated sperm mitochondrial DNA injury, modulated cytochrome B and NADH dehydrogenase 5 contents in sperm samples collected from patients diagnosed with leukocytospermia [197]. In case of cryopreserved semen, CUR supplementation led to an upregulation of GPx4 mRNA expression in human spermatozoa [192], while a higher activity of SOD, CAT and GPx levels were observed in bovine semen [198].

Numerous in vitro reports indicate that CUR is able to decrease peroxidative damage to sperm membranes, most likely because of its ability to scavenge lipid peroxyls before these can attack the membrane lipids [188,189,195]. Nevertheless, a certain discrepancy exists, since a handful of studies [193,194] observed no significant decrease of MDA following CUR administration. As such, it may be arguable that CUR may be able to provide protection to the sperm membranes even without modulating the extent of LPO.

The effects of CUR on ejaculated spermatozoa have been investigated before on different mammalian models, however there are still conflicting results to be taken into consideration, particularly with respect to the exact CUR dosage to be supplemented to semen extenders, since it has been hypothesized on numerous occasions that CUR has the ability to exhibit a dual biological activity: while lower CUR concentrations may protect and stimulate the activity of male reproductive cells, higher CUR doses may exhibit toxic effects on the sperm vitality. Since it has been speculated that CUR has the ability to inhibit protein kinase C, which plays important roles in modulating the flagellar movement of spermatozoa [199], this bioactivity could be associated with possible detrimental effects of CUR on the sperm behavior. Furthermore, it was indicated that CUR may exhibit a selective sperm-immobilizing activity, implying its possible use in modern contraceptives [200].

Available data from in vivo as well as in vitro studies indicate a significant “double-edged sword” activity of CUR. It seems that CUR acts exceptionally well in situations where the vitality of male reproductive structures or cells is already compromised (Figure 5), whereas its use as a supplement under physiological conditions must be treated with caution. As such, the exact dose of CUR for its use in the prevention, management or treatment of male reproductive dysfunction must be thoroughly assessed depending on the species as well as reproductive fitness at the moment of CUR supplementation.

## 7. Rutin

Rutin (quercetin-3-O-rutinoside; RUT) is a flavonoid composed of quercetin and rutinose. The biomolecule is widely present in buckwheat, apple, red beans, as well as beverages such as red wine, black and green tea. RUT is a strong antioxidant and has numerous pharmacological benefits that include anticarcinogenic, cytoprotective, anti-mutagenic, antithrombotic, anti-inflammatory, neuroprotective, vasoprotective and cardioprotective properties [201]. RUT is able to modulate a variety of molecular targets including mitogen-activated protein kinase (MAPK) pathways [202] and the apoptotic machinery (phospho-Bad, cleaved caspase 3 and cleaved Poly [ADP-ribose] polymerase (PARP) [203]. The molecule also exhibits antimycotic properties and appears to be a cholesterol modulator [201]. In vitro data suggest that RUT has a scavenger efficiency and higher antioxidant activity when compared to other biomolecules, as assessed by the DPPH (2,2-diphenyl-1-picryl-hydrazyl-hydrate), and TBA (thiobarbituric acid) assays. This effect may be attributable to the intricate catechol structure of ring B, and the presence of both 7- and 5-hydroxyl groups [204,205].

**Figure 6:**
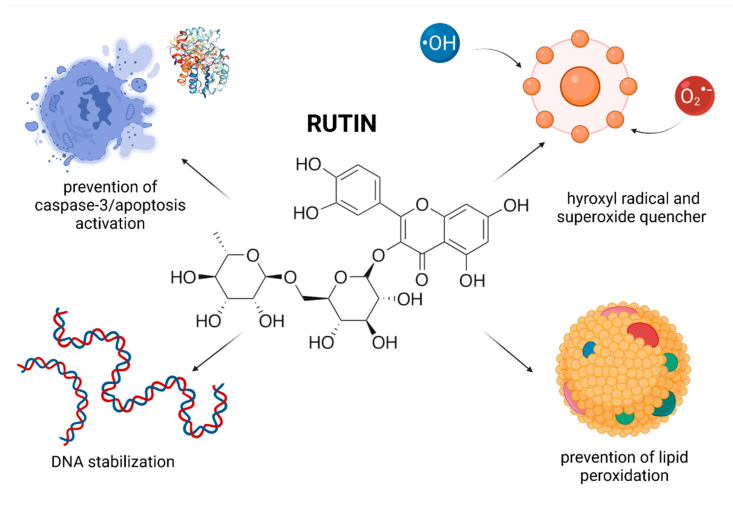
Most frequently reported beneficial effects of rutin on spermatogenesis, sperm structural integrity and functional activity.

According to Raso Becho et al. [206] supplementation of RUT doses up to 20 mg/kg/day manifested itself by a slight reduction of the epididymal weight in the rat, which was reversible at 42 days of completion of treatment. Furthermore, the authors observed no changes in the sperm production throughout the experiment. Neither the seminal vesicles nor the prostate was affected by RUT. As such, no suggestive data of the toxicity of RUT on the reproductive system of adult rats were found, suggesting that its administration is safe for the testicular function (Figure 6).

Protective effects of RUT against reproductive toxicity have been confirmed by a variety of previous studies [207,208,209,210]. Mehfooz et al. [211] found that supplementation of 200 mg/kg RUT was able to ameliorate the testicular histoarchitecture and the process of spermatogenesis in rats subjected to restrain stress. A significantly lower damage to the seminiferous tubules and spermiation arrest were observed as a result of RUT administration. Moreover, RUT was able to prevent the degeneration of round spermatids and vacuolization of the seminal epithelium. Similar protective effects on the structure and function of epididymis and seminal vesicles were observed by Osawe and Farombi [210] in rats subjected to sulphasalazine (SASP). Furthermore, it was noted that RUT is able to inhibit NFκB and TNF-α mediated pathways involved in inflammation [211], to protect mitomycin C induced DNA damage [212] as well as to prevent the cleavage of Poly [ADP-ribose] polymerase 1 (PARP1) and caspase-3 in mouse testes, and thus is able to reduce apoptosis in the testicular tissue [210]. Consequently, RUT was able to restore depleted testicular sperm number and daily sperm production, and increased the sperm morphology and acrosome status in male rats treated with cyclophosphamide and cadmium [207,208,210]. Furthermore, RUT (10 mg/kg) revealed to possess stimulating effects on all sperm parameters including sperm motility, count and viability in Type I diabetic rats [213].

The most plausible mechanism by which RUT exerts its protective functions, is by scavenging excess free radicals (particularly hydroxyl and superoxide radicals), chelating metal ions and reducing LPO levels [201,204,214]. Through these processes, the biomolecule may preserve and enhance testicular and seminal plasma antioxidant enzymes, such as SOD and CAT [205,210,213,215], which then coordinate their activity in protecting developing and functional spermatozoa. Furthermore, it was reported that RUT had a significant beneficial effect on the glutathione cycle, by restoring testicular GSH and GPx activities [210,213,214]. A significant reduction of MDA production was observed in testicular tissue of rodents exposed to gentamycin, cadmium, cisplatin or SASP [205,207,210,215]. Moreover, Akondi et al. [213] observed that RUT reduced testicular LPO in diabetes mellitus by scavenging free radicals generated by hyperglycemia.

The potential of RUT as an antioxidant supplement for the management of male reproductive dysfunction was further fortified by in vitro studies. Mazzi et al. [216] observed that 20 μmol/L and 30 μmol/L RUT were able to preserve human sperm motility, structural integrity and viability. Furthermore, it was demonstrated that RUT had a protective and ROS scavenging effect in sperm cells incubated with TBHP, inductor of LPO. Although, RUT was less active in comparison to QUE, the fact that the molecule contains rutinose, a disaccharide composed of glucose and rhamnose, its ability to penetrate through the plasma membrane is limited, which is why it may be less harmful for sperm function.

RUT has been reported to act as a beneficial and protective supplement for sperm cryopreservation. It was revealed that RUT could prevent cryodamage-inflicted loss of sperm motility in dog [217], ram [218] and red deer spermatozoa [219]. Furthermore, the molecule decreased ROS production and subsequently stabilized the membranes and DNA of cryopreserved spermatozoa.

According to Park et al. [217], 0.1% RUT was more effective in the maintenance of post-thaw progressive motility and viability followed by a stabilization of the oxidative profile of dog spermatozoa in comparison to other antioxidants routinely added to the semen extender (β−mercaptoethanol, GSH or CAT). The authors concluded that RUT can be used as a potentially antioxidative supplement in dog sperm freezing extender based on its ability to modulate gene expression related to motility and apoptosis as well as ROS levels.

## 8. Genistein

Genistein (4′,5,7-Trihydroxyisoflavone; GEN) is a biomolecule belonging to isoflavones, which may be found in soy and fava beans, clover and lupinus. This compound acts as a potent agent in both prophylaxis and treatment of ischemia, atherosclerosis, and cancer. GEN is structurally related to estrogen; and like other phytoestrogens, it can bind to estrogen receptors 1 and 2, and presents with estrogenic as well as anti-estrogenic activities [220,221]. The molecule exhibits significant antioxidant properties, a high affinity towards H_2_O_2_ [220], the ability to inhibit topoisomerase activity, tyrosine kinase signaling pathways and to regulate of both pro- and anti-apoptotic proteins [222]. As such, the advantages of using GEN lie in its multidirectional action and low toxicity of the molecule [220].

**Figure 7:**
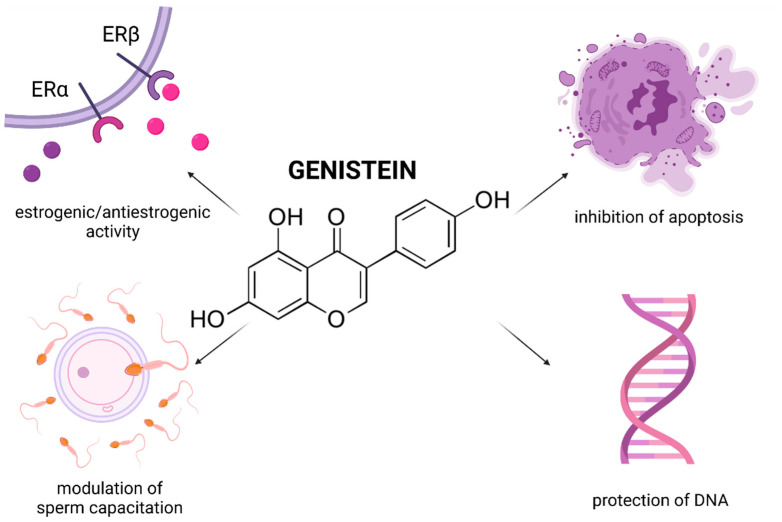
Most frequently reported beneficial effects of genistein on spermatogenesis, sperm structural integrity and functional activity.

Documented evidence suggests that GEN may affect reproductive physiology and function in both animals and humans [223] (Figure 7). It has been proposed that GEN impacts male fertility by either directly targeting testicular function or indirectly by affecting the endocrine activities of the hypothalamic-pituitary-testicular triangular axis. Significant GEN-promoted recovery and improvement in semen quality has been reported by Jalili et al. [224] who observed that GEN (1, 2 and 4 mg/kg/day) enhanced the sperm count in mice treated with morphine, particularly because of its ability to stimulate the proliferation of germinal cells as well as to prevent cell cycle arrest and apoptosis in testicular structures. Furthermore, the authors noted that because of its ROS-trapping abilities, GEN was able to counteract possible oxidative insults to the mitochondrial genome, sperm DNA and membranous structures, leading to the restoration of the production of morphologically and functionally mature spermatozoa with a satisfactory motility and viability.

Furthermore, it has been suggested that GEN may have a direct effect on the function of mature ejaculated spermatozoa, particularly under stress conditions induced by low temperatures. Nevertheless, it must be noted that the exact effect of the biomolecule depends on the species implied in the respective study, the dose of GEN used as well as the exact experimental conditions.

Hinsch et al. [225] focused on GEN as a test substance for routine quality assessments of essential sperm functions, including viability, motility, acrosomal status and inducibility of acrosomal exocytosis, as well as sperm binding to the zona pellucida. It was observed that pre-incubation of bovine cryopreserved swim-up spermatozoa with 0.2 μg/mL GEN led to fewer spermatozoa bound to the zona pellucida, however the molecule had no distinct effect on the sperm motility. It was also revealed that GEN itself did not induce acrosomal exocytosis. The authors assumed that the underlying mechanism may be associated with the ability of GEN to inhibit protein tyrosine kinases and the activity of sodium channels [226], leading to a decreased tyrosine phosphorylation which plays a key role in the process of sperm capacitation and acrosome reaction.

In case of human spermatozoa, Martinez-Soto et al. [227] reported that low GEN concentrations (10 μmol/L) exhibited antioxidant effects on frozen-thawed human spermatozoa, ultimately leading to an improvement in the sperm motility, and a decreased lipid disintegration caused by cryopreservation. A lower DNA fragmentation index observed following GEN supplementation furthermore supports the hypothesis that ROS generation is the most decisive factor endangering a proper condensation and stability of human sperm chromatin following cryopreservation [228]. This suggestion was confirmed by Thomson et al. [229] who indicated that the administration of even higher GEN doses (50 μmol/L or 100 μmol/L) to the cryoprotectant had a significant protective effect on sperm DNA as evaluated by the TUNEL and 8-OHdG assays; in addition to a significant increase of human sperm motility and viability. More recently, Bennetts et al. [230] reported that when fresh human spermatozoa were incubated in the presence of a higher GEN concentration range (31–500 μmol/L) ROS overproduction was detected only in the case of 500 μmol/L GEN. Nevertheless, none of the selected GEN doses affected the sperm viability or DNA integrity. On the other hand, Anderson et al. [231] pointed out that high GEN doses (250 μmol/L) could inflict damage to the sperm DNA integrity similarly to H_2_O_2_ as assessed by the Comet assay. Nevertheless, it must be noted that the experiment was performed with samples from a single donor, while Sierens et al. [232] reported showed that lower GEN concentrations (0.01–100 μmol/L) led to a significantly lower DNA fragmentation in spermatozoa exposed to H_2_O_2_.

Studies on other mammalian species present contradictory results. Kumi-Diaka et al. [233] stated that low doses of GEN (30 μg/mL) did not affect mice sperm motility, however its high concentrations (70 and 100 μg/mL) interfered with the sperm motion behavior. According to Macías-García et al. [234], none of the GEN doses tested (0–800 μmol/L) had any effect in frozen-thawed equine sperm motility or viability. The fact that the addition of GEN did not significantly improve the basic quality parameters of semen led to the assumption that excessive ROS production prior, or during freezing may not be the main factor affecting equine sperm vitality exposed to low temperatures in comparison to cold or osmotic shock.

A different behavior of GEN was observed by Kim et al. [82] on boar sperm characteristics. Sperm motility and viability were increased following 50 μmol/L GEN addition and a subsequent incubation for 3 or 6 h. Furthermore, a beneficial impact of 50 μmol/L GEN was observed in case of the membrane integrity and mitochondrial activity, although it must be noted that these characteristics decreased in the presence of higher GEN concentrations (100 μmol/L). A similar observation was reported by Elsayed et al. [235] who studied the impact of different GEN concentrations (0, 1, 5, 10 and 100 μmol/L) on the freezability and functional integrity of ram spermatozoa. The study reveals that high GEN levels (100 μmol/L) reduced the post thaw motility while low doses of the biomolecule (10 μmol/L) acted as an effective motility-promoting agent. The authors suggest that high GEN concentrations may inhibit the progressive forward motility via alterations to the signal transduction pathways of the sperm cell [233]. Assessment of the sperm viability, functional membrane integrity and acrosomal status complemented the data gathered from the motility analysis and confirmed a clear dichotomy of GEN on the structural integrity and functional activity of male gametes. Moreover, antioxidant properties of the molecule were confirmed by a significantly increased intracellular antioxidant status and a lower oxidative damage to the sperm lipids. Finally, low GEN doses (1 and 100 μmol/L) led to a decline of the caspase-3 mRNA expression suggesting the ability of the biomolecule to inhibit apoptosis as a side-effect of the cryodamage inflicted to spermatozoa [16].

Finally, interesting results were obtained in case of frozen-thawed bovine sperm quality and pronuclear formation after in vitro fertilization (IVF). While 10 μmol/L GEN did not improve neither the sperm motility nor viability, sperm DNA fragmentation was decreased, which may be a valuable insight when using sperm samples with a greater sperm DNA fragmentation index and when motility variables are not important (such as in the case of ICSI) [236].

While being a tyrosine kinase inhibitor, the role of GEN in the capacitation process is still not well defined. Fraser et al. [237] reported that low GEN concentrations (1, 10 and 100 nmol/L) accelerated the capacitation and acrosome loss in human spermatozoa assessed by the chlortetracycline (CTC) staining. On the other hand, several studies have shown the ability of GEN supplemented at a higher concentration to inhibit the progesterone-induced acrosome reaction [238,239]. As such, the exact mechanism underlying the impact of GEN on the capacitation processes deserves further clarification.

Summarizing the above-mentioned studies, both human and animal spermatozoa exposed to high GEN concentrations exhibited some forms of morphological aberrations, abnormal motility, and the loss of fertilizing capability. On the other hand, lower GEN doses seem to improve fertility and offer protection against insults to the male reproductive system. Furthermore, besides differences related to an adequate GEN concentration, another question that needs to be considered, is related to the possibility that different mechanisms are responsible for induced capacitation of fresh or cryopreserved spermatozoa. Indeed, in case of frozen bull or equine spermatozoa, the biomolecule did not affect tyrosine phosphorylation or the acrosome status [234,240,241], suggesting that the process of cryopreservation per se affects the regulatory mechanisms of capacitation.

## 9. Apigenin

Apigenin (4,5,7-trihydroxyflavone; API) is a flavone subclass of flavonoid which is abundantly present in aromatic plants (rosemary, chamomile, parsley), vegetables and fruits (apple, celery, fennel) as well as other foodstuffs (honey) [242]. The molecule has attracted a widespread attention because of its estrogenic activity, anti-inflammatory [243], anticancer [244], anti-diabetic [245] and antioxidant properties [246]. It has been proposed that the antioxidant behavior of API stems from its ability to scavenge free radicals and chelate metal ions [247]. An important aspect of API lies in its ability to be taken up by cells easily and to bind to DNA via both intercalation and external binding, which forms API-DNA complexes and protects DNA from oxidative stress within cells [242,248].

Currently available in vivo studies suggest that API exerts effects against some poisons that cause injuries in animals [249,250,251]. Dang et al. [250] revealed that API was able to prevent degeneration of the mitochondrial midpiece and outer dense fibers of the flagellum of epididymal spermatozoa exposed to acrylonitrile, critical for a proper epididymal transit or ejaculation. Furthermore, the study reported that API presented with the ability to attenuate acrylonitrile-induced testicular apoptosis of the spermatogenic cells and later spermatozoa by restoring the intracellular balance between anti-apoptotic and pro-apoptotic regulators is critical to cell survival, and the ratio of Bcl-2/Bax, which was later confirmed by the TUNEL assay.

Akilah et al. [251] chose to study the effects of API on a rat model of male infertility caused by chloroquine. In parallel with the previous study, the authors noted a decreased caspase-3 immunohistochemical expression, a decreased rate of apoptosis, followed by a prevention of the reduction of sperm count, viability, and testicular weight. Further experiments revealed that API could protect against ischemia-reperfusion and heat-induced testicular damage through its antiapoptotic activity [252,253].

**Figure 8:**
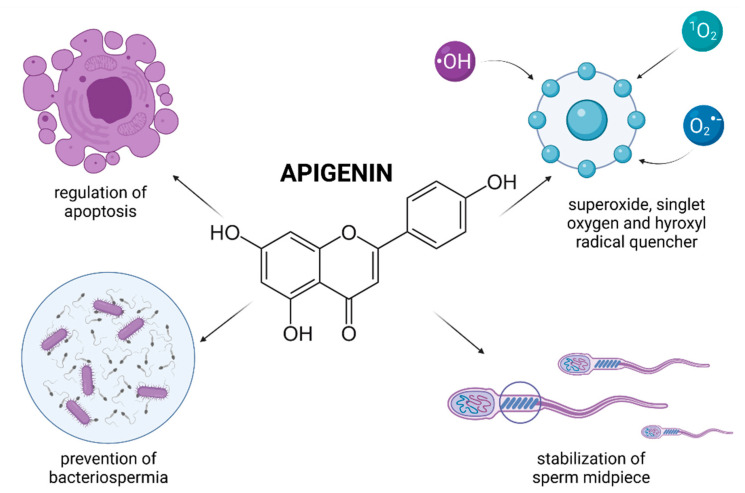
Most frequently reported beneficial effects of apigenin on spermatogenesis, sperm structural integrity and functional activity.

Although being less active than its homologue genistein, the inherent toxicity of API is extremely low [242], which may make it an ideal supplement for sperm cell processing, culture, and storage. This hypothesis was confirmed by several studies on cryopreserved bovine and boar semen (Figure 8).

According to Pei et al. [254], the motility, mitochondrial function, membrane and acrosomal integrity of cryopreserved boar sperm increased with the addition of API and reached their peaks at 0.2 mmol/L API. Then these parameters stayed stable or even dropped significantly when higher doses of API were added to the extender. The authors noted that API was exceptionally effective for the sperm to acquire better mitochondrial activity, confirming the ability of API to trespass relatively freely through the sperm cell, and thus exhibit its activities intracellularly and have a better protective effect on the mitochondrial membrane. What is more, Wang et al. [255] confirmed that API could reduce bacterial contamination and improve the kinematic parameters and abnormal semen movement in frozen semen as a consequence of bacteriospermia.

API has been proven to be a potent antioxidant with the ability to eliminate superoxide, singlet oxygen, and hydroxyl radicals in vitro and to protect and/or enhance the intracellular antioxidant defense system. Previous in vivo research reported that API could restore both enzymatic (SOD, CAT, and GPx) as well as non-enzymatic (GSH, vitamin C and E) antioxidant systems [242].

According to Dang et al. [250] API was able to restore the glutathione cycle, crucial for the protection of the structure and function of the cell membrane against peroxides, and failure of which may be considered to be an early event in the apoptotic cascade that kinetically could precede the loss of mitochondrial integrity [256]. Furthermore, the authors reported that API might have a protective effect against LPO of the spermatogonial cells and epididymal spermatozoa caused by free radicals. Similarly, Akilah et al. [251] noted that API decreased the testicular MDA level, raised the testicular GSH level and CAT activity. An enhancement of SOD and GPx activity and decreased testicular MDA concentration was reported in heat-induced testicular damage in rodents [252].

Similar observations have been recorded in case of spermatozoa. Pei et al. [254] reported that the intracellular SOD and CAT activities of the post-thawed boar spermatozoa were significantly increased, and the MDA levels were significantly decreased by adding 0.2 mmol/L API. The same API concentration ensured a lowed ROS and MDA generation in frozen bull spermatozoa, while simultaneously enhancing SOD activity and GSH levels.

Nevertheless, as with other biomolecules, the optimal concentration of API supplemented must be monitored closely. As speculated by Li et al. [257] API concentrations as high as 25 mg/kg may have adverse effects on the reproductive system in adult male mice, as evidenced by a lower sperm density, the occurrence of abnormal spermatozoa and cellular arrest in the seminiferous epithelium.

API is an active constituent of *Striga orobanchioides*, a medicinal plant with contraceptive properties [258]. Among the potential mechanisms to explain these effects, the ability of API to interact with estrogen receptors [242], enhancement of gap-junction intercellular communication, and apoptosis have been proposed [259]. Furthermore, caution is advised with respect to API supplementation to the sperm processing media, as in case of a high dose of API, this permeable molecule may alter the osmolality of the extender, and subsequently cause aberrations to the sperm structure and function [254].

## 10. Naringenin

Naringenin (NAR; 2,3-dihydro-5,7-dihydroxy-2-(4-hydroxyphenyl)-4H-1-benzopyran-4-one) is a natural flavonoid belonging to flavanones, and is commonly found in citrus fruits, tomatoes, bergamot, cherries, and cocoa. Recently, the molecule has been abundantly studied for its antiproliferative, anti-inflammatory, and antimutagenic activities [260]. Furthermore, it has been shown to act as a carbohydrate metabolism promoter [261], immunity modulator [262], and DNA stabilizing agent [263]. NAR is considered a safe natural product with an estrogenic and/or partial anti-estrogenic activity [260] and significant antioxidant properties.

Evidence gathered from animal studies suggests that NAR is highly efficient in attenuate a compromised testicular function and associated sperm functionality following exposure to medicaments [264], oxidative agents [265] or may serve as a prophylactic agent to manage testicular dysfunction associated with a medical condition [266]. Mostafa et al. [267] reported that NAR could protect from permethrin-induced testicular alterations such as a decreased testicular weight and the epididymal sperm count. Similar observations were reported by Adana et al. [264] in the case of rats exposed to highly active antiretroviral therapy and by Sahin et al. [265] who studied the effects of NAR on H_2_O_2_-administered rats. Furthermore, 50 mg/kg NAR administration led to a restoration of the mineral profile of the testicular milieu and prevented histopathological changes in the testicular structure such as sloughing and detachments following exposure to H_2_O_2_.

A relatively lower dose of NAR (5 mg/kg and 10 mg/kg) was selected by Roy et al. [266] to counteract male reproductive dysfunction in STZ-diabetic rats. According to the authors, NAR-treated rats in the diabetic group showed improved histological and sperm parameters, along with a decrement of terminal dUTP nick end-labeling (TUNEL) detected cell death and a reduced overexpression of proinflammatory molecules. These results suggest that NAR could act as anti-inflammatory and anti-apoptotic agent in male reproductive structures.

According to Adana et al. [264] 40 and 80 mg/kg NAR proved potent enough to attenuate the highly active antiretroviral therapy (HAART)-induced toxicity on spermatozoa as evidenced by a significantly higher sperm count, motility, morphology, and DNA integrity. The authors speculate that this positive impact of NAR on the male gametes could lie in the ability of NAR to scavenge ROS and prevent excessive copper and iron interactions. It has been also proposed that NAR could restore mitochondrial membrane potential mitigating mitochondrial function and prevent a subsequent initiation of the apoptotic cascade [267]. The data collected from the Comet assay also revealed that NAR had the ability to protect the sperm DNA from disintegration, leading to the assumption that NAR could induce DNA repair enzymes or upregulate the antioxidant mechanisms to neutralize ROS produced by HAART.

The ability of NAR to modulate the redox balance in favor of a balanced state is furthermore evidenced by a restoration of the testicular levels of SOD, GPX and GSH levels accompanied by a significant reduction of LPO in animals subjected to H_2_O_2_ or STZ toxicity [265].

NAR has proven to be an effective supplement to media aimed for sperm storage or cryopreservation. According to Tvrdá et al. [81], exposure of extended boar spermatozoa to 10 and 25 mol/L NAR led to a maintenance of sperm motility for up to three days of storage. Similar data were collected in previous studies on chilled and/or frozen rooster and boar semen [268,269], suggesting that NAR may have stimulating effects on the motion behavior because of its lipophilic and antioxidant properties that enable the molecule to protect the membranous structures of the male gamete, including the plasma membrane and acrosome. Furthermore, it was noted that NAR has mitochondrial-protecting properties, which may manifest themselves in a stabilized mitochondrial membrane potential, activity of succinate dehydrogenase, a reduced quantity of mitochondrial ruptures and a lower risk for the activation of the mitochondrial-mediated apoptotic pathway. According to Mehdipour [269], a more balanced ratio of pro-apoptotic Bax/anti-apoptotic Bcl-2 gene expression patterns was observed in cryopreserved rooster spermatozoa following exposure to NAR, which was accompanied by a reduced release of cytochrome C and caspase-3 activation [270].

**Figure 9:**
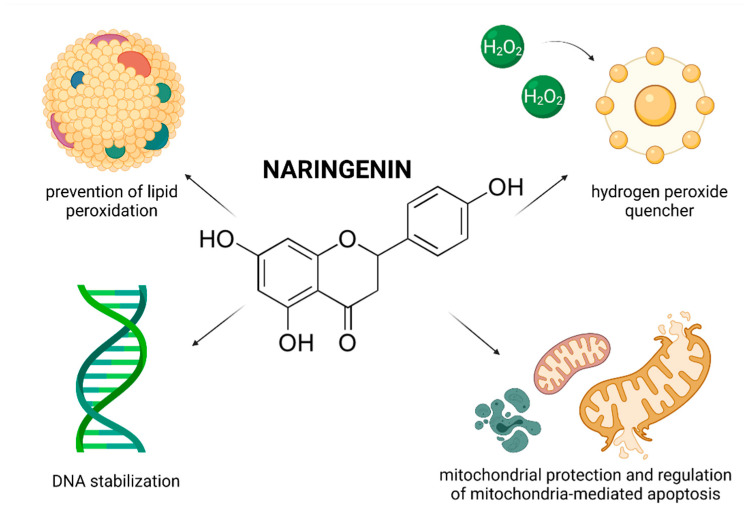
Most frequently reported beneficial effects of naringenin on spermatogenesis, sperm structural integrity and functional activity.

Several studies imply that NAR is a highly effective ROS quencher, able to diffuse into the membranes and is able to interfere particularly with H_2_O_2_ [265]. It has been proposed that NAR is able to neutralize excessive H_2_O_2_ in the sperm membranes, which will become unavailable for further intracellular conversion to the hydroxyl radical, and thus sperm DNA damage may be prevented. Furthermore, NAR exhibited a strong ability to inhibit peroxidative damage to the sperm lipids. This property may be directly associated with the polar nature of NAR, which may facilitate its adherence to the lipid bilayer, and thus prevent oxidative insults to the sperm cell membrane [271]. Summarizing the gathered information, it may be hypothesized that NAR acts as a global ROS quencher, leading to a decreased LPO and a subsequent plasma membrane, acrosome, and DNA stabilization, which has a direct impact on the sperm motion (Figure 9).

According to Mehdipour [269], NAR supplementation to frozen rooster spermatozoa leads to a higher population of useful spermatozoa in the sperm storage tubules, crucial for a successful sperm penetration to the inner perivitelline layer and avian fertility [272]. As such, NAR may be effective for spermatozoa to accomplish their journey within the hen reproductive tract to reach sperm storage tubules and subsequently an optimal fertilization position, which will result in a higher number of fertilized eggs and hatching rates.

As with other biomolecules, NAR may exhibit prooxidant properties under certain circumstances. As suggested by Ranawat and Bakshi [273] NAR can act as a prooxidant by undergoing peroxidase-catalyzed oxidation of the phenol B ring present in its molecule. The phenoxyl radicals which are produced from oxidized NAR, may subsequently co-oxidize GSH to form thiyl radicals. According to the authors, inappropriate doses of NAR uncover its pro-oxidant properties, subsequently causing damage to the testicular tissue, a decreased sperm concentration and motility.

This dichotomy of NAR may be observed in vitro as well, as revealed by Tvrdá et al. [81] where NAR concentrations above 50 μmol/L had deteriorating effects on the maintenance of boar sperm motion and mitochondrial stability. A similar observation was recorded by Moretti et al. [94], who noted that NAR concentrations below 200 μmol/L had a generally favorable effects of effect on the progressive motility and viability of human swim-up sperm, while a sharp decline in the sperm vitality was observed following administration 200 μmol/L and 400 μmol/L NAR. As such, the exact dosage of NAR must be carefully assessed, depending on the aims and conditions of the experiment.

## 11. Kaempferol

Kaempferol (3,4′,5,7-tetrahydroxyflavone; KAE) has only recently emerged as a molecule with a promising biological activity which may be found primarily in leafy vegetables (kale, leeks, spinach) and herbs (dill and chives). Currently, studies on the beneficial properties of KAE are primarily focused on its cardioprotective effects and anti-inflammatory activity, while a potential toxicity or adverse events of KAE intake have not been confirmed [274,275].

**Figure 10:**
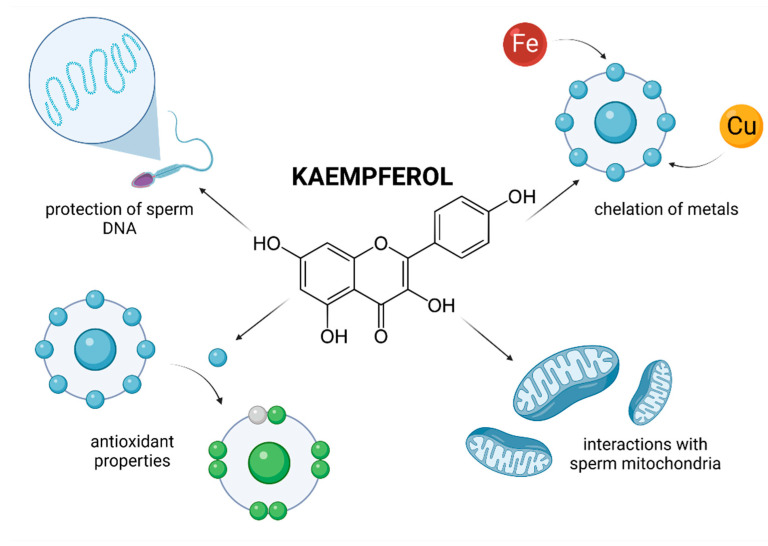
Most frequently reported beneficial effects of kaempferol on spermatogenesis, sperm structural integrity and functional activity.

Currently, only a handful of reports have studied the in vitro effects of KAE on the sperm survival (Figure 10). The molecule has shown to act a potent flavonoid in the protection of male gametes exposed to toxicosis induced by lead, cadmium, or aluminium. The molecule showed the highest efficiency amongst all tested biomolecules in the sperm motility recovery as well as in the prevention of LPO, based on which it may be hypothesized that besides its antioxidant properties, the molecule is able to interfere with the motion apparatus and/or the mitochondrial system of spermatozoa [160]. This hypothesis may be further fortified by the results obtained by Ďuračka et al. [268] who observed a significant preservation of sperm motility and mitochondrial activity in stored boar semen in the presence of KAE even after 72 h. A significantly decreased ROS production in semen extended in the presence of KAE may provide further evidence to its antioxidant properties, and thus a decreased oxidative damage to the sperm membranes. The exact mechanism of action however needs to be explored further. 

Another possible mechanism of action by which KAE prevents sperm deterioration is protection of the DNA molecule against damage and strand breakage as shown by Anderson et al. [276]. In their study, the authors revealed protective and antigenotoxic effects of KAE on the sperm DNA integrity using the Comet assay. KAE alone produced a positive response in the sperm in comparison with the control group, however this protective effect became less potent when human spermatozoa were exposed to food mutagens such as 3-amino-1-methyl-5H-pyrido(4,3-b)indole (Trp) and 2-amino-3-methylimidazo-(4,5-f)quinoline (IQ). Different behavioral patterns of KAE in response to different risk factors of the food chain need to be the focus of further studies, although it must be remembered that while semen samples from 24 donors were used in the study by Jamalan et al. [160], semen from only one subject was studied by Anderson et al. [276].

Although preliminary data show a promising potential of KAE as a protective and antioxidant molecule, it has been suggested that high amounts of KAE supplementation may cause self-oxidation [277,278]. On the other hand, animal studies found that no pro-oxidation was present following oral intake of KAE [279]. Furthermore, it is noteworthy to evaluate the hypothesis that KAE may decrease iron absorption and folic acid uptake because of its reactivity with these nutrients [279,280].

## 12. General Observations

Summarizing evidence gathered from the available literature, we may conclude that each natural biomolecule affects the sperm production and subsequent behavior through a complex intracellular array of actions that is unique and specific to each natural compound. Furthermore, it must be noted that each biomolecule presents with an inherent dichotomy—while the administration of a correctly selected dose comes along with a wide spectrum of favorable effects on the process of spermatogenesis and sperm vitality, high levels of these may lead to numerous adverse effects that will manifest themselves in a deterioration of male fertility [14,17,18,32]. As such, Table 1 summarizes the principal molecular mechanisms of action by which the selected biomolecules manifest their beneficial effects on male reproductive tissues and cells. The range of concentrations in which these beneficial properties occur in vivo or in vitro is indicated as well.

By and large, all presented biomolecules may be characterized by significant antioxidant properties that may be helpful in restoring the balance between ROS generation and inherent ROS-scavenging properties of antioxidant enzymes present in the seminal plasma [3,24,29]. Since oxidative stress has become a notorious pathology observed in sub- or infertile men, the assessment of selected markers of oxidative damage to the male gametes should the first step in the treatment of male infertility through the administration of natural biomolecules. Such primary assessment of the extent and/or individual parameters contributing to seminal oxidative stress could be helpful in the selection of the most suitable biomolecule, its optimal dosage and timing in the context of counteracting of ROS-inflicted damage to spermatozoa.

## 13. Conclusions

In conclusion, this review aimed to summarize currently available information on the effects of natural biomolecules on the spermatogenesis, structural integrity as well as functional activity of male gametes. By and large, natural compounds exhibit positive roles on male fertility, primarily through their antioxidant, antiapoptotic and DNA protecting properties, their ability to stimulate mitochondrial metabolism and hence sperm motility and fertilizing ability. Nevertheless, since also harmful effects of natural biomolecules have been observed in the context of male fertility, and the exact mechanisms underlying this behavior have only recently started to be investigated more thoroughly, a critical approach is advised before drawing definite conclusions on potential benefits or adverse effects of phytochemicals on the male reproductive system. Since a compound exhibiting a specific biological effect may not be the same native molecule found in natural resources, further studies are necessary to assess the activity of adjacent metabolites as opposed to just the native biomolecules that are currently in the scientific spotlight. At the same time, it must be remembered that in vivo, most molecules may act in synergy or antagony depending on the conditions, which needs to be considered in the interpretation of their in vitro behavior. Finally, establishment of a normal physiological range of biomolecules such as polyphenols, flavonoids or carotenoids in reproductive fluids and tissues is a critical prerequisite that may assist to determine if the effects achieved from a certain dose in an experimental study are physiologically relevant, since a proportion of scientific studies may employ doses that exceed normal physiologic levels. Accordingly, all these aspects should be taken into consideration in future studies providing more clarification to the positive or negative effects of natural biomolecules on the sperm production and function.

## Figures and Tables

**Table 1:** Principal mechanisms of action and recommended concentrations of selected biomolecules for in vivo and in vitro administration.

Biomolecule	Molecular Mechanism of Action	Concentration Range Exhibiting Beneficial Effects In Vivo	Concentration Range Exhibiting Beneficial Effects In Vitro
Resveratrol	Inhibition of ROS overproduction and ROS-scavenging properties. Stabilization of sperm membranes through the prevention of LPO. Modulation of mitochondrial metabolism through SIRT1 and AMPK-regulated pathways. Modulation of specific paternal transcripts and spermatogenic regulatory proteins.	20 mg/kg/day (physiological conditions) [38]	5–25 µmol/L (human sperm) [48,50,51,53]
		20–50 mg/kg/day (pathological conditions) [42,43,44,45,46]	15–50 µmol/L (animal sperm) [49,54]
Quercetin	Regulation of the expression levels of antioxidant enzymes and inflammatory factors. Modulation of the activity of CatSper channels and Ca^2+^-ATPase. Regulation of the Ca^2+^-influx, cAMP concentration and ATP production. Superoxide trapping abilities through the inhibition of NADPH oxidase/NADH-dependent oxidoreductase and SOD mimicking.	90–270 mg/kg/day (physiological conditions) [69]	10–30 µmol/L (human sperm) [79,94]
		25–50 mg/kg/day (pathological conditions) [70,71,72,73]	10–25 µmol/L (animal sperm) [80,81,82,83,84,85,86,87]
Lycopene	Stabilization of the hydrophobic core of membranous structures. Active scavenging of singlet oxygen, hydroxyl radical and nitrogen dioxide. Prevention of oxidative damage to sperm DNA.	14 mg/day (physiological conditions) [108]	5 µmol/L (human sperm) [130]
		4–30 mg/day (pathological conditions) [111,113,121,124,125]	0.5–1.8 mmol/L (animal sperm) [127,128,129,130,133]
Catechins	Chelation of trace and heavy metals. Prevention of Fenton and Haber-Weiss reaction. Modulation of estrogen receptors, cholesterol efflux and tyrosine phosphorylation. Stimulation of adenylate cyclase/cAMP/PKA signaling; lipase and G6PDH activity. Synergistic activity with GPx.	50 mg/kg/day [141,142]	2–25 µmol/L (human sperm) [94,154,160]
			25–100 µmol/L (animal sperm) [147,148,149,150,151,152]
Curcumin	Modulation of cytochrome B/P450 isoenzymes and NF-κB DNA-binding activity. Stabilization of sperm chromatin, specifically lysine-rich histones, chromatin condensation, cytosine-rich and guanosine sequences. Inhibition of the Fenton reaction, xanthine oxidase and nitric oxidase activity. Upregulation of mRNA expression levels of SOD, CAT and GPx4.	30–150 mg/kg/day (physiological conditions, depending on the animal species) [166,167]	20 µmol/L (human sperm) [192]
		20–100 mg/kg/day (pathological conditions) [168,170,171,172,173,174,176,178]	5–50 µmol/L (animal sperm) [188,189,191,193,196]
Rutin	Inhibition of NFκB and TNF-α mediated pathways involved in inflammation. Modulation of the cleavage of Poly [ADP-ribose] polymerase 1 (PARP1) and caspase-3. Regulation of the glutathione cycle.	20 mg/kg/day (physiological conditions) [206,209]	20–30 µmol/L (human sperm) [216]
		10–30 mg/kg/day (pathological conditions) [205,207,208,210,212,213]	1.0–1.5 mmol/L (animal sperm) [217,218,219]
Genistein	Modulation of estrogen receptors. Stabilization of the mitochondrial membrane and mtDNA. Modulation of the activity of protein tyrosine kinases and sodium channels.	4 mg/kg/day [224]	10–100 µmol/L (human sperm) [227,229,232]
			10–50 µmol/L (animal sperm) [16,82,235,236,237]
Apigenin	Protection of sperm DNA via API-DNA complexes formed through intercalation or external binding. Modulation of estrogen receptors and Bcl-2/Bax expression levels. Regulation of gap-junction intercellular communication, and apoptosis.	15–30 mg/kg/day [250,251,252,253]	0.2 mmol/L [254,255]
Naringenin	High affinity to lipid membranous bilayers. Inhibition of oxidative chain reactions via direct H_2_O_2_ quenching. Modulation of pro-apoptotic Bax/anti-apoptotic Bcl-2, cytochrome C and caspase-3 gene expression patterns.	50–80 mg/kg/day [264,265,267]	25–100 µmol/L [81,268,269]
Kaempferol	Metal chelation. Interactions with molecular pathways of detoxification. Inhibition of genotoxicity.	N/A	500 µmol/L (human sperm) [277,278]
			10–50 µmol/L (animal sperm) [268]
